# An auxin-mediated ultradian rhythm positively influences root regeneration *via EAR1/EUR1* in *Arabidopsis*

**DOI:** 10.3389/fpls.2023.1136445

**Published:** 2023-06-07

**Authors:** Quy Thi Vu, Kitae Song, Sungjin Park, Lin Xu, Hong Gil Nam, Sunghyun Hong

**Affiliations:** ^1^ Center for Plant Aging Research, Institute for Basic Science, Daegu, Republic of Korea; ^2^ Department of New Biology, Daegu Gyeongbuk Institute of Science and Technology (DGIST), Daegu, Republic of Korea; ^3^ National Key Laboratory of Plant Molecular Genetics, CAS Center for Excellence in Molecular Plant Sciences, Institute of Plant Physiology and Ecology, Chinese Academy of Sciences, Shanghai, China

**Keywords:** ultradian rhythm, auxin, excised Arabidopsis leaf, EAR1/EUR1, *de novo* root regeneration

## Abstract

Ultradian rhythms have been proved to be critical for diverse biological processes. However, comprehensive understanding of the short-period rhythms remains limited. Here, we discover that leaf excision triggers a gene expression rhythm with ~3-h periodicity, named as the excision ultradian rhythm (UR), which is regulated by the plant hormone auxin. Promoter–luciferase analyses showed that the spatiotemporal patterns of the excision UR were positively associated with *de novo* root regeneration (DNRR), a post-embryonic developmental process. Transcriptomic analysis indicated more than 4,000 genes including DNRR-associated genes were reprogramed toward ultradian oscillation. Genetic studies showed that EXCISION ULTRADIAN RHYTHM 1 (EUR1) encoding ENHANCER OF ABSCISIC ACID CO-RECEPTOR1 (EAR1), an abscisic acid signaling regulator, was required to generate the excision ultradian rhythm and enhance root regeneration. The *eur1* mutant exhibited the absence of auxin-induced excision UR generation and partial failure during rescuing root regeneration. Our results demonstrate a link between the excision UR and adventitious root formation *via EAR1/EUR1*, implying an additional regulatory layer in plant regeneration.

## Introduction

Biological rhythms are ubiquitous periodic cycles that impact the physiology, behavior and transcriptome of living organisms and play critical roles in their ecological fitness (McClung, 2011; Laje et al., 2018). In addition to well-known circadian (~ 24-h) rhythms, ultradian (< 24-h) rhythms, despite a much smaller number of studies (Prendergast and Zucker, 2016), have been reported to be critical for diverse biological processes in both animals and plants such as development, cell fate decision and metabolism (Aulehla and Pourquié, 2008; Moreno-Risueno et al., 2010; Isomura and Kageyama, 2014; Zhu et al., 2017). In plants, the most notable ultradian rhythm (UR) is the cytosolic calcium oscillations in guard cells, which is required for stomatal closure (McAinsh et al., 1995; Staxén et al., 1999; Allen et al., 2000). In *Arabidopsis thaliana*, periodic root branching is accompanied by a ~6-h gene expression rhythm and disruption of this rhythmic gene expression impairs root formation (Moreno-Risueno et al., 2010). Ultradian rhythms display an enormous diversity in periodic phenomena with various frequencies and characteristics (Wollnik, 1989; Goh et al., 2019); therefore, origin and biological significance of ultradian rhythms remain opened questions and many biological processes which may be associated with ultradian rhythms have not been identified yet. Here, we serendipitously discovered a ultradian gene expression rhythm in excised *Arabidopsis* leaves and investigated the physiological relevance and the genetic mechanism underlying the occurrence of this rhythm.

The ability to regenerate organs and tissues, particularly after predator attack or mechanical wounding, is a critical survival mechanism that allows organisms to replace or augment lost or damaged organs and tissues. Recent studies revealed extensive molecular regulatory mechanisms underlying regeneration in animals and plants, and highlighted their potential application in regenerative medicine and agriculture (Stoick-Cooper et al., 2007; Ikeuchi et al., 2019). Plants possess a unique and remarkable regeneration capability. Plant regeneration not only replaces lost tissue and organs, but can also lead to the genesis of new organs and even entire organisms (Ikeuchi et al., 2016). This unique capability, given their sessile lifestyle, allows plants to optimize survival and propagation under hostile ecological situations (Lup et al., 2016). Genesis of new organs from organ explants or *de novo* organogenesis allows plants to develop new organs such as shoots or roots from excised parts of plants, and this is frequently observed in nature (Xu and Huang, 2014). Excised *Arabidopsis* leaves, which can regenerate adventitious roots (ARs) at the excision site on a hormone-free medium, are frequently used as a model system to imitate natural conditions and investigate the molecular mechanisms underlying *de novo* root regeneration (DNRR) (Chen et al., 2014). DNRR is a highly complex process that involves time-evolving regulatory networks with a series of cell fate transition, division and differentiation steps which require reprogramming of large set of gene expression to generate ARs at final step (Xu, 2018; Jing et al., 2020). Despite recent extensive studies, the regulatory mechanisms of gene expressions underlying the DNRR process are not fully understood.

Here we discovered EAR1 as a key regulator to activate a UR in excised leaves. Our study also suggests a new regulatory layer that the UR triggered by leaf excision resets gene expression patterns, thereby assisting the cells at the excision sites to reorient from their predetermined differentiated cellular states toward new fates to optimize adventitious root regeneration.

## Materials and methods

### Plant materials and growth conditions

*Arabidopsis thaliana* ecotype Columbia (Col-0) was used as wild-type control. The transgenic *CCA1::LUC* (Salomé and McClung, 2005), *CAB2::LUC* (Millar et al., 1992), *ORE1::LUC* (Kim et al., 2018), *DR5::LUC* (Moreno-Risueno et al., 2010) and *ARF7::LUC* (Moreno-Risueno et al., 2010) lines have previously been described. *PRR7::LUC* was kindly donated by Xie and McClung. To generate the *PIN3::LUC, AFB2:LUC* and *KMD1::LUC*, 2,031 bp of *PIN3*, 2,078 bp of *AFB2* and 2,014 bp of *KMD1* promoter regions were cloned into the pZPXomegaLUC vector (Schultz et al., 2001) to fuse with the firefly luciferase gene and they were introduced into Col-0 plants by Agrobacterium tumefaciens-mediated transformation. The transgenic *ORE1::LUC* in *eur1-14* (SALK_108025C), *yuc5/8/9* (CS69942) and *yuc2/5/8/9* (CS69869) were generated by Agrobacterium tumefaciens-mediated transformation. For complementation of *eur1-11* mutant, *EAR1::EAR1-GFP* was constructed by fusing a combination of full length genomic DNA fragment without stop codon and 2,092 bp of promoter fragment of *EAR1* in front of Green Fluoresent Protein (GFP) in the gCogn-eGFP-N-1300 (Cambia, Canberra, Australia). To generate EAR1^141-287^ fragment-harbouring transgenic lines on *eur1-11* mutant, *EAR1::EAR1^141-287^-FLAG* was constructed by Gateway cloning method and transformed to *eur1-11* mutant by Agrobacterium tumefaciens-mediated transformation. See **Supplementary Table 2** for primer details. *Arabidopsis thaliana* plants were grown in an environmentally controlled growth room (Korea Instruments, Korea) at 22°C under 16 h light/8 h dark conditions with ~100 µmol m^-2^s^-1^ white light. For confocal microscopic assay, *eur1-11/EAR1::EAR1-GFP* seeds were surface-sterilized with 1% sodium hypochlorite for 10 min and rinsed 4 times with sterilized distilled water. After 3 days in cold condition, sterilized seeds were planted on half strength B5 medium (Duchefa, The Netherlands) containing 0.8% agar (type M; Sigma) with 1% sucrose and grown in the same condition.

### Luminescence assay

Transgenic plants expressing luciferase under control of various gene promoters were used in this assay. Whole seedlings, or indicated samples were excised from transgenic plants and transferred to 48- or 24-well microplates containing 5.7 pH of 3 mM MES (2-(N-morpholino) ethanesulfonic acid, Amresco, USA) solution with 500 µM luciferin (SYNCHEM, Felsberg/Altenburg, Germany). Those plates were put in luminescence chambers under continuous light (~20 µmol m^-2^ s^-1^) condition at 22°C. Luminescence images were acquired every 30 min with 5-min exposure times for at least 3 days and images were analyzed with the MetaView system (Molecular Devices, USA).

### Wavelet analysis for detecting rhythms

The luminescence intensities were quantified by continuous wavelet transformation techniques, which are implemented into Wavelet Comp package in R (Rösch and Schmidbauer, 2016) for detecting circadian rhythm (CR) and ultradian rhythm (UR). The rhythms were detected by periodic parameters of 2 to 6 hours for UR and 16 to 32 hours for CR.

### *De novo* root regeneration assay

For root regeneration assay, the 4^th^ rosette leaves were excised from indicated ages of plants and placed on half strength B5 medium (Duchefa, The Netherlands) (pH 5.7) containing 0.8% agar without sucrose. To prevent fungal contaminations, we added plant preservative mixture (PPM) (Plant cell technology, Washington, USA) with ratio 1:3,000. The plates were cultured under continuous light conditions with ~20 µmol m^-2^s^-1^ white light at 22°C. The number of adventitious roots from leaf explants was determined by counting the regenerated root tips in the petiole regions during the indicated day. The rooting rate was calculated as the ratio of root tip regeneration to total cultured leaf explants on the indicated day. The rooting images were taken using a SMZ1500 stereomicroscope (Nikon, Japan), with 0.75× objective.

### Sampling to identify UR oscillating genes

The 4^th^ rosette leaves from soil-grown 21 day-old plants were excised at their petiole by forceps and floated in plates containing 3 mM MES solution (pH 5.7). Plates were incubated under dark and 16°C for 24 hours and then transferred to ~20 µmol m^-2^s^-1^ continuous white light condition at 22°C. Leaves were collected at 16 different time points between 19 and 27 hours after pre-treatment. Total cellular mRNA was extracted from WelPrep (Welgene, Daegu, Korea). Contaminating DNA was removed by digestion with DNase I (DNA-*free*™ Kit DNase Treatment and Removal, Invitrogen, Thermo Fisher Scientific), then RNA quality was assessed on an Agilent BioAnalyzer 2100. RNA integrity numbers (RINs) for the samples were calculated and found the average RIN to be 7.5 with which many mRNA-sequencing experiments have been performed.

### Sampling for RNA-seq to identify DEGs between wild-type and *eur1-11*

The 4^th^ rosette leaves from soil-grown 21 day-old plants of Col/*ORE1::LUC* as wild-type and *eur1-11* mutant were excised at their petiole by forceps and floated in plates containing 3mM MES solution (pH 5.7). Plates were incubated under ~20 µmol m^-2^s^-1^ continuous white light conditions at 22°C. Petiole regions of excised leaves were collected at 0, 24, 48, 72 and 96h since detachment. Total mRNA was extracted from leaves using WelPrep (Welgene, Daegu, Korea). Contaminating DNA was removed by digestion with DNase I (DNA-*free*™ Kit DNase Treatment and Removal, Invitrogen, Thermo Fisher Scientific), then RNA quality was assessed on an Agilent BioAnalyzer 2100.

### RNA-seq and functional prediction

Library construction and sequencing were performed using Illumina Hiseq 2500 platform for detecting oscillation genes and using Illumina NovaSeq 6000 platform for eur*1-11*. Raw reads were checked quality and trimmed using FastQC (Andrews, 2010), and the trimmed reads were mapped to the Arabidopsis thaliana genome (TAIR10) using STAR (Dobin et al., 2013). After alignment, the gene-level raw count data files were generated using HTSeq (Anders et al., 2015) and normalized using edgeR’s TMM algorithm (Robinson et al., 2010). The differential gene expression was analyzed by the multifactor generalized linear model (GLM) approach in edgeR with replicate number added as a factor to the GLM to mitigate for a batch effect. The filtered genes with a *p*-value under 0.05 were considered as differential expressed genes. Gene ontology (GO) and Kyoto Encyclopedia of Genes and Genomes (KEGG) pathway were performed by g:Profiler for computing multiple hypothesis testing corrections (g:scs < 0.05) (Reimand et al., 2007). The ReViGO was used to summarize and visualize the list of significantly enriched GO terms based on semantic similarities (allowed similarity: 0.5) (Supek et al., 2011). The RNA-seq data used in this study have been deposited in the Gene Expression Omnibus (GEO: http:/www.ncbi.nlm.nih.gov/geo/) and assigned the identifier accession GSE157230 and GSE158133.

### Detecting oscillation genes

The RNA-seq dataset for UR detection (synchronized RNA-seq dataset) was analyzed by bioinformatics tools (described above). The MetaCycle R package which incorporates ARSER, JTK CYCLE and Lomb-Scargle (Wu et al., 2016) was used to detect rhythmic genes from Synchronized RNA-seq data. The ultradian rhythms were detected with parameters: minper 2 h and maxper 5 h. The genes with cut-off (*p*-value < 0.05) based on meta2d results were defined as UR oscillating genes.

### Clustering for differential expressed genes in *eur1-11*

The expression values of DEGs were analyzed by the tri-cluster system, TimesVector (Jung et al., 2017) for the relationship between time series and pattern of DEGs.

### ABA seed germination and primary root growth assay

The seeds were sown on half strength B5 medium (Duchefa, The Netherlands) containing 2% (w/v) sucrose and 0.8% agar, incubated under a 16 h light/8 h dark cycle with a light intensity of ~100 μmol m^−2^ s^−1^ light at 22°C in the environmentally controlled growth room. For the seed germination assay, 30 seeds were sown on half strength B5 medium containing different concentration of ABA. The plates were incubated under a 16 h light/8 h dark cycle with a light intensity of ~100 μmol m^−2^ s^−1^ light at 22°C in the environmentally controlled growth room for 8 days to examine the seed germination ratio. For the primary root growth assay, 6 day-old seedlings were transferred to vertical plates of half strength B5 medium containing different concentrations of ABA and grown for an additional 4 days. The plates were then scanned by HP Scanjet 8300 and primary root length was measured by ImageJ program.

### EMS Mutagenesis and Screening assay for the UR regulators

Approximately 20,000 seeds (M1) of Col-0/*ORE1::LUC* line were mutagenized by treatment with 0.3% or 0.33% ethyl methanesultonate (EMS) solution for 8 hours. M2 seeds were obtained by self-fertilization of the M1 plants. M2 seeds were sown on half strength B5 medium (Duchefa, The Netherlands) containing 1% sucrose and 0.8% agar (pH 5.7). The plates were placed under 16h light/8h dark (LD) with ~100 μmol m^−2^ s^−1^ light at 22°C in the environmentally controlled growth room until 2 weeks-old. The 1^st^ or 2^nd^ leaves were excised by forceps at the petiole base and transferred to 96-well microplates containing 500µM luciferin (SYNCHEM, Felsberg/Altenburg, Germany). Luminescence images were acquired every 30 min for at least 3 days under continuous white light conditions at 22°C by CCD camera. 16 experimental runs were done to check UR patterns in excised leaves from ~16,000 M2 plants. 175 M2 plants whose excised leaves not showing the UR were selected as candidates for the UR regulators. Because the UR was fragile and sensitive with aging, we only selected plants completely removing the UR with maintaining green after leaf excision across UR measuring time as Col-0/*ORE1::LUC* parent. These selected plants were moved to soil and grew for M3 seeds. We after that re-checked UR pattern in excised leaves with at least 8 M3 plants of each selected mutant lines. 28 mutant lines in M3 were confirmed with UR disappearance in excised leaves. Among 28 lines, we finally found 4 homozygous lines not showing the UR in excised leaves and selected those lines as candidates of the UR regulators for further investigation.

### Genomic DNA library preparation for whole genome sequencing (WGS)

For WGS, pools of genomic DNA were prepared from 20-25 seedlings of 14 day-old plate-grown Col/*ORE1::LUC* as control and *eur1-11*, *eur1-13* backcrossed with Col/*ORE1::LUC* F2 not showing the UR in excised leaves. After grinding samples in the liquid nitrogen, genomic DNA was extracted by CTAB (cetyltrimethyl ammonium bromide) extraction buffer containing 100 mM Tris-HCl (pH 8.0), 20 mM EDTA (pH 8.0), 1.4 M NaCl, 2% (W/V) CTAB and 1% PVP 40, 000 (polyvinyl pyrrolidone) and mixture of phenol/chloroform/isoamyl alcohol (25:24:1) (Thermo Scientific). Contaminating RNA was removed by adding 2.5µl of RNAse A (10 mg/ml) (Roche) in every 500µl of CTAB extraction buffer. Genomic DNA products finally were purified by QIAGEN Dneasy Plant Mini Kit.

### Whole genome sequence and SNP detection in EMS mutants

The DNA library construction from EMS mutants was prepared using Truseq Nano DNA Prep Kit (Illumina, San Diego, CA, USA). The quality was verified by Agilent 2100 Bioanalyzer (Agilent, Santa Clara, CA, USA), then the passed libraries were loaded onto NovaSeq 6000 Sequencing system (Illumina, San Diego, CA, USA) by DNALink, South Korea (https://www.dnalink.com), as instructed in the manufacturer’s protocols. The reads were quality checked and filtered by fastp(Chen et al., 2018). The clean reads were aligned to the reference genome of TAIR10 and genetic variants were called according to SIMPLE pipeline (Wachsman et al., 2017).

### Microscopic assay

To confirm EAR1 protein levels in petiole region, the 1^st^ or 2^nd^ rosette leaves were excised from plate grown 14-day-old plants. The leaf explants were cultured on half strength B5 medium without sucrose and incubated under continuous light conditions with ~20 µmol m^-2^s^-1^ white light at 22°C. For confocal laser scanning microscopy, samples at indicated time were observed using a Zeiss LSM 7 DUO system (Carl Zeiss), with a 20 x objective. Wavelengths used to visualize GFP and autofluorescence of chloroplasts were 500-540 and 600-640 nm, respectively. Tiled images were taken with ZEN software (Carl Zeiss) and processed with Adobe Photoshop.

## Results

### Leaf excision evokes an auxin-mediated UR

**Figure 1:**
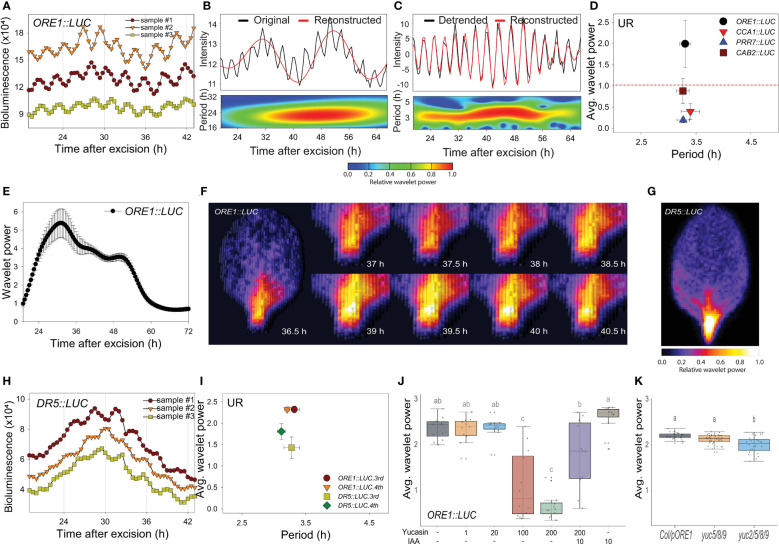
An excision-triggered ultradian rhythm is mediated by auxin in *Arabidopsis* leaves. **(A)** *ORE1* promoter activity in *Arabidopsis* leaves at the indicated time points. The graph shows three representative samples. At least three different experiments were performed with similar results. **(B, C)** Wavelet analysis-based decomposition of *ORE1* promoter activity showing **(B)** circadian rhythm (CR) and **(C)** ultradian rhythm (UR). Black and red lines (upper panels) represent luminescence intensity and reconstructed rhythm, respectively. Wavelet spectrum plots (lower panels) indicate period range and wavelet power through the indicated time period. Red and blue indicate higher and lower wavelet powers, respectively. **(D)** Average wavelet powers and periods of UR in the activities of *ORE1*, *CCA1*, *PRR7* and *CAB2* promoters, as quantified by wavelet analysis. Data are means ± s.e.m. (*n* = 24 leaves). Red line indicates the UR threshold. **(E)** Instantaneous UR wavelet power of *ORE1* promoter activity in *ORE1::LUC* leaves. The wavelet power for UR shows the distribution of UR through the indicated time period. Data are means ± s.e.m. (*n* = 24 leaves). **(F)** Time-series analysis of *ORE1::LUC* expression in the petiole region after leaf excision. **(G)** Image showing *DR5::LUC* luminescence in an excised leaf. **(H)** *DR5::LUC* activity in excised Arabidopsis leaves at the indicated time points. The graph shows three representative samples. **(I)** Average wavelet powers and periods of UR in *ORE1* and *DR5* promoter activities in excised leaves, as determined by wavelet analysis. Data are means ± s.e.m. (*n* = 12 leaves). **(J)** Average wavelet powers of UR in excised leaves expressing *ORE1::LUC* treated exogenously with yucasin or indole-3-acetic acid (IAA) (*n* = 12 leaves). **(K)** Average wavelet powers of UR in excised leaves from wild-type, *yuc5/8/9* and *yuc2/5/8/9* mutants expressing *ORE1::LUC* (*n* = 24 leaves). Centre line: median; bounds of box: 25th and 75th percentiles; whiskers: 1.5 × IQR from 25th and 75th percentiles. Statistical significance was determined by one-way analysis of variance (ANOVA) with Tukey’s *post hoc* test. Data points with different letters indicate statistically significant differences between groups (*P* < 0.01).

We previously used the firefly *luciferase* (*LUC*) reporter gene (Kim et al., 2016; Kim et al., 2018) to track the promoter activities of circadian clock-regulated genes in transgenic *Arabidopsis*, including *ORE1* (*ORESARA 1*). The *ORE1* gene was initially identified as a positive regulator of leaf aging in *Arabidopsis* (Kim et al., 2009). Later, the gene was found to be under circadian transcriptional and post-transcriptional regulatory control through a circadian clock component, PRR9 (Kim et al., 2018). The 3^rd^ or 4^th^ rosette leaves were excised from 21-day-old plants grown under long-day conditions (16 h light/8 h dark), and LUC activity was monitored at 30 min intervals using a CCD camera under continuous white light at 22°C (**Supplementary Figure 1A**). Transgenic *ORE1::LUC* leaves showed short period rhythms (**Figure 1A**). We used wavelet analysis, which is suitable for time-frequency data (Leise, 2013), to determine whether periodicity resulted from an endogenous biological rhythm. As the *ORE1* promoter exhibited a circadian rhythm (CR) as well as UR, we separated the circadian component by reconstructing the smoothed circadian signal from the original oscillating pattern. Consequently, the wavelet spectrum exhibited a ~24 h period CR (**Figure 1B**). Subtraction of the CR wavelet from the original oscillating pattern revealed an additional UR with a ~3 h period (**Figure 1C**). Using wavelet analysis, we also calculated average UR wavelet power which was defined as mean of UR wavelet power of all tested samples along time scale as in **Figure 1C**. *ORE1*, *CCA1* (*CIRCADIAN CLOCK ASSOCIATED 1*), *PRR7* (*PSEUDO-RESPONSE REGULATOR 7*) and *CAB2* (*CHLOROPHYLL A/B-BINDING PROTEIN 2*) promoter activities in excised leaves showed various wavelet powers (**Figure 1D** and **Supplementary Figures 1B-I**). When a threshold of 1.0 was established to discriminate the UR from noise (**Figure 1D**, red line), *ORE1* promoter activity showed a significant wavelet power. We further characterized this ~ 3 h rhythm using *ORE1* promoter activity.

We used wavelet analysis of *ORE1* promoter activity to determine whether the UR was present in intact leaves and other excised organs. Attached leaves did not exhibit a UR in *ORE1* promoter activity (**Supplementary Figures 2A–C, N**). Next, we examined *ORE1* promoter activity in 7-day-old whole seedlings and in excised shoot apices, cotyledons, hypocotyls and roots (**Supplementary Figure 2D**). The UR was detected in excised cotyledons but not in the other samples (**Supplementary Figures 2E–I, N**). We also tested organs excised from bolted plants (**Supplementary Figure 2J**). The UR was present in excised cauline leaves but not in excised flowers or stems (**Supplementary Figures 2K–N**). Thus, the *ORE1* UR is leaf specific and is evoked upon its excision. The ~3 h period UR was therefore named the ‘excision UR’.

To gain insight into the physiological function of the excision UR, we examined its temporal and spatial expression patterns in excised leaves. A significant excision UR wavelet power was observed from ~19 h after excision and maintained until ~ 60 h before dampening over time (**Figure 1E**). The excision UR thus functioned as a transient response to excision. The highest level of *ORE1* promoter activity with robust oscillations was observed in the petiole region close to the excision site (**Figure 1F**). By contrast, *ORE1* promoter activity persisted across the whole area of an attached leaf (**Supplementary Movie 1**). The localized and transient nature of the UR proximate to the excision site supported the conclusion it was a response to leaf excision.

Excised *Arabidopsis* leaves undergo a drastic developmental shift toward *DNRR* at the excision site. By ~12 h after excision, auxin is produced in converter cells and transported to the vasculature near the wound site, where it is involved in further DNRR processes (Chen et al., 2016; Bustillo-Avendaño et al., 2018; Xu, 2018; Jing et al., 2020). These studies suggested the excision UR might be related to the production of auxin as an excision response. To test this, we monitored auxin responses *in vivo* and in real time using *DR5::LUC* transgenic plants (Moreno-Risueno et al., 2010). DR5 is a synthetic promoter harboring auxin response elements (AuxREs) which can be bound by auxin response factors (ARFs) (Ulmasov et al., 1997). DR5 promoter activity is driven by interaction between ARFs, Aux/IAA and auxin level. *DR5::LUC* expression exhibited an excision UR with a significant wavelet power (**Figures 1H, I**) and luciferase activity was enriched in the petiole region (**Figure 1G**). This result indicates that auxin biosynthesis and signaling pathways, at some levels, are controlled by the excision UR. On the other hand, the average wavelet power of the *ORE1* promoter excision UR was reduced by yucasin, an auxin biosynthesis inhibitor (Nishimura et al., 2014), and rescued by exogenous auxin (**Figure 1J**). To further support the role of auxin in excision UR, we generated *ORE1::LUC* transgenic lines on auxin biosynthesis mutants. Endogenous auxin in plants is majorly biosynthesized by tryptophan (Trp)-independent and tryptophan-dependent pathways (Cao et al., 2019). In Arabidopsis, *YUCCA* gene family contains 11 members and catalyses conversion of indole-3-pyruvate acid (IPyA) to indole-3-acetic acid (IAA) in Trp-dependent pathway. YUCCA-mediated auxin biosynthesis was also known to involve in *de novo* root organogenesis in excised *Arabidopsis* leaf (Chen et al., 2016). *yuc2/5/8/9* quadruple mutant, but not *yuc5/8/9* triple mutant, significantly reduced the average value of *ORE1* promoter excision UR (**Figure 1K**) which may be due to auxin action. There are no clear morphological defects in *yuc5/8/9* and *yuc2/5/8/9* mutants but *yuc2/5/8/9* shows more severely reduced fertility (Müller-Moulé et al., 2016). The results support that auxin is required for the UR generation in excised leaf. Taken together, the data indicate that auxin and the excision UR exert reciprocal control at petiole region of excised leaves.

### Excision UR positively correlates with DNRR

**Figure 2:**
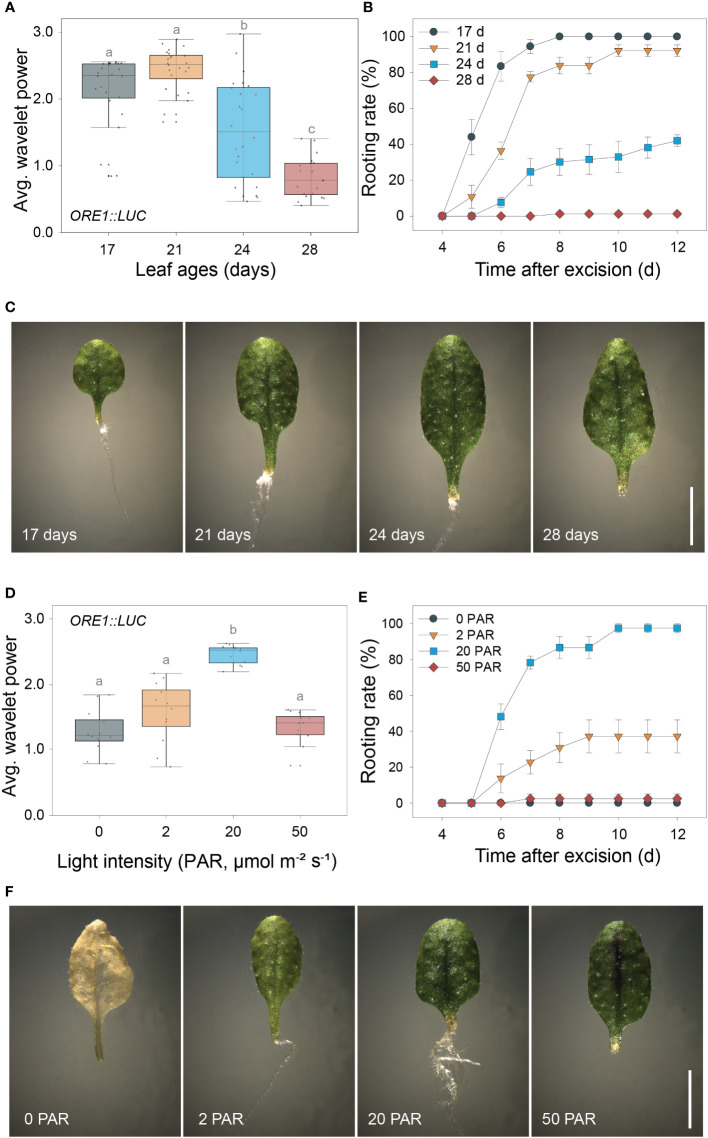
Positive correlation between the excision UR and *de novo* root regeneration (DNRR). **(A)** Average wavelet powers of the excision UR in wild-type leaves excised from plants of different ages (*n* = 24 leaves). **(B)** Rooting rates of wild-type leaves of different ages. Data are means ± s.e.m. from three independent replicates. **(C)** Images showing root regeneration 10 days after excision in wild-type leaves of different ages. Scale bar: 5 mm. **(D)** Average wavelet powers of the excision UR in wild-type leaves exposed to different light intensities (*n* = 12 leaves). In **(A, D)** centre line: median; bounds of box: 25th and 75th percentiles; whiskers: 1.5 × IQR from 25th and 75th percentiles. Statistical significance was determined by one-way analysis of variance (ANOVA) with Tukey’s *post hoc* test. Data points with different letters indicate statistically significant differences between groups (*P* < 0.01). **(E)** Rooting rates in wild-type leaves exposed to different light intensities. Data are means ± s.e.m. from three independent replicates. **(F)** Images showing root regeneration from wild-type leaves under different light intensities.

As both the excision UR and DNRR were induced by leaf excision and controlled by auxin, we assessed the relationship between robustness of the excision UR and efficiency of DNRR in excised leaves under various conditions. DNRR is highly sensitive to the age of an excised leaf (Chen et al., 2014; Pan et al., 2019), with aged leaves exhibiting a marked reduction in DNRR capacity. The excision UR and DNRR efficiency were examined in leaves excised from plants of different ages. The average wavelet powers of the excision UR were robust in the 4^th^ leaf from 17- or 21-day-old plants, but gradually decreased with leaf age; the excision UR was not detectable in leaves from 28-day-old plants (**Figure 2A**). The efficiency of DNRR was positively correlated with the trend in excision UR (**Figures 2B, C**). Thus both the excision UR and DNRR were highly sensitive to leaf age, and occurrences of the two events were correlated.

DNRR is sensitive to light conditions, as excised leaves form roots under light conditions but not in the dark without sucrose (Chen et al., 2014). We therefore examined the effect of varying light intensity on excision UR robustness and DNRR efficiency. The average wavelet powers of the excision UR were highest under photosynthetically active radiation (PAR) of 20 µmol m^-2^ s^-1^, and were reduced in the dark and under lower or higher light intensities (**Figure 2D**). Similarly, DNRR was most efficient under PAR of 20 µmol m^-2^ s^-1^ (**Figure 2E**). Excised leaves did not produce any roots under darkness due to dark-induced senescence, a rapid ageing process (**Figure 2F**). Leaves exposed to lower and higher light intensities remained green for 12 days after excision but showed reduced DNRR efficiency (**Figures 2E, F**). Excision UR robustness was therefore positively correlated with DNRR efficiency under various light intensities.

### Function and expression of a large set of excision UR genes

**Figure 3:**
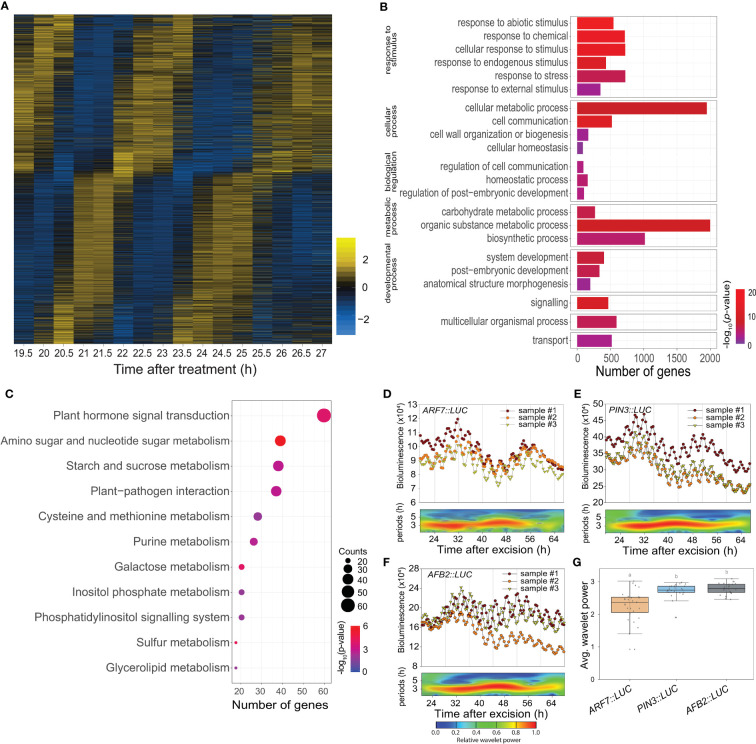
Transcriptomic and functional analysis of the excision UR genes. **(A)** Heat map showing the expression levels of genes oscillating over time. Yellow and blue indicate higher and lower relative expression, respectively. **(B)** Gene ontology enrichment analysis of the excision UR genes. Bars represent numbers of genes and color represents the *p* value. **(C)** KEGG enrichment analysis of the excision UR genes. Dot size indicates the number of genes, and dot colour represents the *P*-value. **(D-F)** Analysis of *ARF7, PIN3* and *AFB2* promoter activities using the *LUC* reporter. Graphs show data from three representative samples. The graphs (upper panels) show measurements from three representative samples (n = 24) and the wavelet spectrum plots (lower panels) show merged wavelet power plots of all samples with low transparency. **(G)** Average wavelet powers of the excision UR of *ARF7, PIN3* and *AFB2* (*n* = 24 leaves). Centre line: median; bounds of box: 25th and 75th percentiles; whiskers: 1.5 × IQR from 25th and 75th percentiles. Statistical significance was determined by one-way analysis of variance (ANOVA) with Tukey’s *post hoc* test. Data points with different letters indicate statistically significant differences between groups (*P* < 0.01).

Time-lapse transcriptome analysis was used to examine the physiological roles of the excision UR further. We used MetaCycle analysis, an established method for evaluating periodicity in time-series data (Wu et al., 2016), to identify genes involved in the excision UR at the transcriptional level. Expression of 4,073 genes oscillated with period lengths between 2.9 and 4.3 h (**Figure 3A** and **Supplementary Dataset 1**), indicating that a relatively large set of genes displayed a ultradian oscillation in response to leaf excision. Gene Ontology (GO) analysis revealed these genes encompassed a broad range of biological processes. The GO terms ‘responses to stimuli’, ‘metabolic process’ and ‘developmental process’ were enriched (**Figure 3B** and **Supplementary Dataset 2**). A further enrichment analysis using the Kyoto Encyclopedia of Genes and Genomes (KEGG) database (**Figure 3C**) found ‘plant hormone signal transduction’ (KEGG:04075) was the most strongly enriched pathway, as 61 excision UR genes were annotated in this pathway (**Supplementary Dataset 2**). Several metabolic pathways were also enriched among the excision UR genes. Genes acting in hormone signal transduction pathways and multiple metabolic processes were enriched and reset toward ultradian oscillation, thus, suggesting that the excision UR might predominantly function in those processes.

DNRR at the leaf excision site involves a complex array of regulatory genes. Auxin plays an essential role in this process (Chen et al., 2014; Chen et al., 2016; Bustillo-Avendaño et al., 2018; Xu, 2018; Jing et al., 2020): of 40 DNRR-associated genes identified previously, twelve, of which seven were auxin-related, showed the UR expression pattern (**Supplementary Table 1**). The excision UR thus regulated the expression patterns of genes involved in auxin-related DNRR. The effect of the excision UR on auxin-related genes was confirmed using promoter-reporter assays. LUC activity in transgenic plants expressing *PIN-FORMED 3* (*PIN3*)*::LUC*, *ARF7::LUC* or *AUXIN SIGNALING F-BOX 2* (*AFB2*)*::LUC* showed robust excision UR (**Figures 3D–G**).

Auxin also regulates a root clock, which produces oscillations in gene expression with a ~6 h period for prebranch site production (Moreno-Risueno et al., 2010). We compared the genes showing ~3 h period UR with microarray data from the root clock to determine whether these different URs shared common molecular components. The two datasets showed little overlap (< 7%). Among that, *YUCCA 9* (*YUC9*) and *AUXIN RESPONSIVE FACTOR 7* (*ARF7*) were common to both (**Supplementary Figure 3**). Both are auxin-related genes involved in DNRR, suggesting that, although the two URs controlled distinct sets of genes, they shared part of the auxin-mediated regulatory pathways.

### EAR1, an abscisic acid (ABA) signaling component, positively regulates the excision UR to optimize DNRR

**Figure 4:**
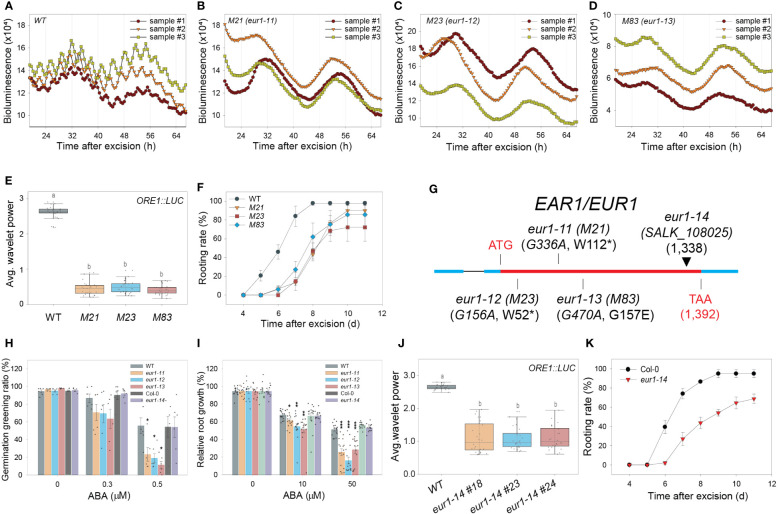
EAR1/EUR1, an abscisic acid signaling component, regulates the excision UR to optimize DNRR. **(A-D)** *ORE1* promoter activity in *ORE1::LUC* transgenic plants and EMS-mutagenized mutant candidates. **(A)** wild type (WT); **(B)** *M21*; **(C)** *M23*; **(D)** *M83*. Graphs show data from three representative samples. **(E)** Average wavelet powers of the excision UR in WT plants and mutant candidates (*n* = 24 leaves). **(F)** Rooting rates of WT plants and mutant candidates. Data are means ± s.e.m. from three independent replicates. **(G)** Schematic representation of the mutation sites in *eur1-11* (*M21*), *eur1-12* (*M23*), *eur1-13* (*M83*) and *eur1-14* (SALK_108025). **(H, I)** Effects of treatment of *Col/ORE1::LUC* (background of *eur1-11, 1-12* and *1-13* mutants) used as WT, *eur1-11*, *eur1-12*, *eur1-13*, *Col-0* (background of *eur1-14*) and *eur1-14* plants with exogenous ABA on germination greening ratio (*n* = 6) **(H)** and relative root growth **(I)** (*n* = 13 seedlings). Two-tailed t-test was used between wild type and *eur1* mutants (*p ≤ 0.05, **p ≤ 0.01, ***p ≤ 0.001). **(J)** Average wavelet powers of the excision UR of wild type, *eur1-14* #18, *eur1-14* #23 and *eur1-14* #24 (*n* = 24 leaves). **(K)** Rooting rates of Col-0 and *eur1-14*. Data are means ± s.e.m. from three independent replicates. Statistical significance was determined by one-way analysis of variance (ANOVA) with Tukey’s post hoc test. Data points with different letters indicate statistically significant differences between groups (P < 0.01).

A forward genetic screen was performed to search for genetic factors involved in excision UR generation and/or function. Transgenic *ORE1::LUC* seeds were mutagenized with ethyl methane sulfonate (EMS). Leaves excised from individual M_2_ plants were screened for the absence of the excision UR. Four homozygous lines (*M21*, *23*, *38* and *83*) were identified after screening ~16,000 M_2_ plants (**Figures 4A–E** and **Supplementary Figure 4A, B**). Genetic analyses revealed that all four candidates were recessive mutants. *M21*, *M23* and *M83* belonged to the same complementation group, whereas *M38* formed a second distinct complementation group (**Supplementary Figure 4C**). The mutations were named *EXCISION ULTRADIAN RHYTHM* (*EUR*), and the first and second complementation groups were named *EUR1* and *EUR2*, respectively. All four mutants exhibited delayed initiation of ARs relative to wild type (**Figure 4F** and **Supplementary Figure 4D**), supporting that the excision UR is upstream of AR formation during DNRR process, as the four mutants belonged to two independent complementation groups, and yet controlled the excision UR and AR initiation simultaneously. This is consistent with temporal order in appearance of the excision UR and AR emergence (**Figure 1E** and **Figure 2B**) during DNRR process (Jing et al., 2020).

The presence of three *eur1* mutant alleles in one complementation group facilitated molecular analysis by whole-genome sequencing (WGS). The WGS data of *ORE1::LUC* (parental line) were compared with that of a pool of F_2_ homozygous mutant progeny, which showed no excision UR, obtained by backcrossing *M21* or *M83* with *ORE1::LUC*. Only one gene, *ENHANCER OF ABSCISIC ACID (ABA) CO-RECEPTOR1* (*EAR1*), harboured common intragenic single nucleotide polymorphisms (SNPs) in both the *M21* and *M83* mutants (**Supplementary Figure 4E**). The WGS results were validated by sequencing the *EAR1* coding sequence in *M21*, *M83* and *M23* (**Supplementary Figure 4F**). In *M21* and *M23*, tryptophan residues at amino acid positions 112 and 52 were changed to nonsense codons, whereas glycine-157 was changed to glutamate in *M83* (**Figure 4G**). The mutant alleles in *M21*, *M23* and *M83* were named *eur1-11*, *eur1-12* and *eur1-13*, respectively. To confirm that *EAR1* was the gene responsible for the excision UR, complementation lines (*COM-9*, *COM-24*) were generated by expressing an *EAR1-GFP* fusion construct under the control of its cognate promoter (*EAR1::EAR1-GFP*) in the *eur1-11* mutant background. The expression of *EAR1::EAR1-GFP* rescued both the impaired excision UR and delayed AR initiation phenotypes of *eur1-11* (**Supplementary Figure 5**). Furthermore, to confirm that *EAR1* is key regulator of the overall excision UR, not only *ORE1* excision UR, we generated *Luciferase* transgenic lines driven by several promoters of UR oscillating genes like *PIN3*, *AFB2* and *KMD1 (KISS ME DEADLY 1)* on wild-type and *ear1-1* background (Wang et al., 2018). The excision UR of these promoter activities was entirely gone in *ear1-1* mutant (**Supplementary Figure 6**). Taken together, these results indicated that *EAR1* corresponded with the *eur1* mutations and was a positive regulator of excision UR in excised *Arabidopsis* leaves.

EAR1 is a negative regulator of ABA signaling, and the EAR1^141-287^ fragment is sufficient for EAR1 function in ABA responses (Wang et al., 2018). We tested whether the fragment of EAR1/EUR1 generated the UR in a similar manner by using an insertion line of *EAR1* (SALK_108025, *eur1-14*), in which the T-DNA is inserted at position 1,338 of *AT5G22090* (**Figure 4G**), keeping the fragment intact. Unlike the other *eur1* mutant alleles, ABA responses, inhibition of germination and root growth in *eur1-14* resembled those of wild-type plants (**Figures 4H, I**), confirming previous reports (Wang et al., 2018). Notably, however, both expression of the excision UR and DNRR efficiency were impaired in *eur1-14* leaves (**Figures 4J, K**), suggesting that EAR1/EUR1-mediated excision UR generation and AR formation are separate from canonical ABA signaling. To further support this conclusion, we generated transgenic lines harbouring EAR1^141-287^ fragment driven by its own promoter on *eur1-11* mutant background. Although those transgenic lines rescued hypersensitive response to ABA of *eur1-11* mutant, the excision UR entirely could not be recovered while DNRR efficiency was only partially rescued (**Supplementary Figure 7**), implying the contribution of the excision UR to AR regeneration and supporting that EAR1 regulates “the excision UR – AR formation” axis in a separate pathway from canonical ABA pathway. In addition, the data suggest that canonical ABA signalling pathway also regulates DNRR in a UR-independent pathway.

### Auxin-induced generation of the excision UR *via* EAR1/EUR1 enhances root regeneration

**Figure 5:**
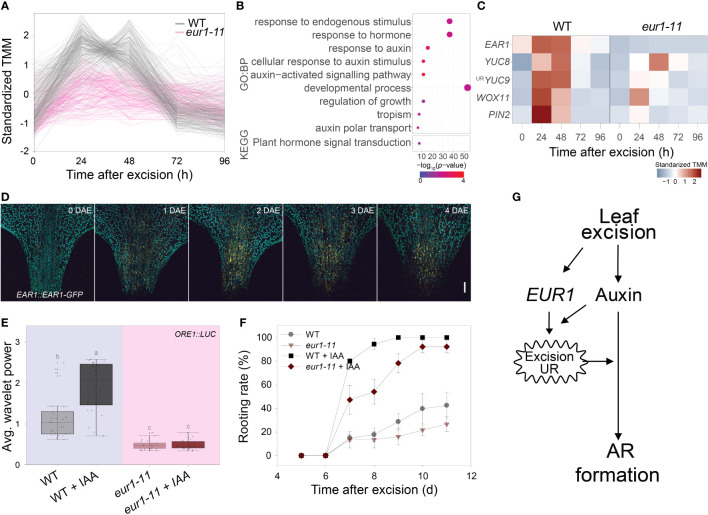
EAR1/EUR1 mediates generation of the auxin-induced excision UR to enhance AR formation. **(A)** Expression patterns of cluster 2 genes, which include *EAR1/EUR1*. **(B)** Gene ontology (GO) enrichment analysis of genes in cluster 2. Dot size indicates the number of genes, and dot colour indicates the *P-*value. **(C)** Heat map of DNRR-associated genes belonging to cluster 2. Expression values from RNA-seq were standardized to allow comparison. **(D)** Tiled confocal images of the petiole region of *COM-9* (*EAR1::EAR1-GFP*) leaves. Yellow indicates EAR1-GFP fluorescence; blue indicates chlorophyll autofluorescence. Two independent lines were analysed with similar results. Scale bar: 0.2 mm. **(E, F)** Effect of IAA treatment on average wavelet powers of the excision UR **(E)** (*n* = 24 leaves) and rooting rates of wild-type and *eur1* leaves excised from 24-day-old plants **(F)**. In **(E)** centre line: median; bounds of box: 25th and 75th percentiles; whiskers: 1.5 × IQR from 25th and 75th percentiles. Statistical significance was determined by one-way analysis of variance (ANOVA) with Tukey’s *post hoc* test. Data points with different letters indicate statistically significant differences between groups (*P* < 0.01). In **(F)** data are means ± s.e.m. from three independent replicates. **(G)** Schematic showing EAR1/EUR1-mediated excision UR generation and AR formation in excised leaves.

As the EAR1/EUR1 controlled both the excision UR and AR formation, we investigated the link between these two phenomena. We performed time-course RNA-seq analysis of the petiole regions of wild-type and *eur1-11* mutant leaves collected at 0, 24, 48, 72 and 96 h after excision. This revealed that 9,754 genes were differentially expressed between wild type and *eur1-11*. These differentially expressed genes (DEGs) were categorized into 12 clusters according to the similarity between their expression profiles (**Supplementary Figure 8** and **Supplementary Dataset 3**). Interestingly, the expression profiles of genes in cluster 2, which contained *EAR1/EUR1*, resembled the pattern of excision UR wavelet power (**Figure 5A**). To gain further insight into the role of EAR1/EUR1 in DNRR, we performed GO and KEGG enrichment analyses of the 325 genes belonging to cluster 2. These genes were strongly enriched in GO/KEGG terms related to auxin and development (**Figure 5B**), suggesting that EAR1/EUR1 enhanced AR formation *via* an auxin-mediated developmental processes. Indeed, the DNRR-associated genes found in cluster 2 included key genes required for auxin biosynthesis and transport, and for auxin-mediated cell fate transition, such as *YUC8*, *YUC9*, *PIN2* and *WUSCHEL-RELATED HOMEOBOX 11* (*WOX11*) (Chen et al., 2016; Xu, 2018) (**Figure 5C**). The absence of EAR1/EUR1 altered expression of auxin-related genes in the petiole region upon excision, which may have changed the expression of genes involved in cell fate determination and resulted in delayed AR formation. DNRR occurs at the site of excision from the petiole. Excision UR expression was the strongest at the petiole, which correlated positively with DNRR. We therefore examined the spatial and temporal regulation of EAR1/EUR1 in *EAR1::EAR1-GFP* plants. The fluorescence signal was absent in the petiole region at 0 days after excision (DAE), but was visible from 1 DAE and most abundant at 2 DAE (**Figure 5D**), indicating that the changes in EAR1/EUR1 levels coincided with expression of the excision UR.

Exogenous application of auxin rescues DNRR in aged leaves (Chen et al., 2014). As the excision UR was also regulated by auxin (**Figures 1J, K**) and reduced in aged leaves (**Figure 2A**), we hypothesized that auxin might induce the excision UR through EAR1/EUR1 and rescue DNRR efficiency in aged leaves. To test this, we applied 10 µM IAA to 4^th^ rosette leaves excised from aged (24-day-old) wild-type and *eur1-11* mutant plants, and measured robustness of the excision UR and DNRR efficiency. Exogenous auxin treatment rescued the excision UR wavelet power in aged wild-type leaves but not in aged *eur1-11* leaves (**Figure 5E**), indicating that EAR1/EUR1 was required for auxin-induced excision UR generation. The DNRR efficiency of aged wild-type leaves was also fully rescued by auxin; however interestingly, aged *eur1-11* mutant leaves showed rescued, but still delayed AR initiation compared with wild-type counterparts (**Figure 5F**). In order to examine whether the partial failure in rooting initiation under IAA treatment of *eur1-11* mutant is due to less sensitivity to auxin, we conducted auxin assay and measured root elongation under exogenous IAA treatment. Similar to previous publication that primary root elongation is inhibited by exogenous auxin (Cheng et al., 2004), 0.2µM IAA strongly reduced primary root elongation in *Col/ORE1::LUC* and *eur1-11* in a similar pattern while *axr1-3*, an auxin resistant mutant, showed significantly longer roots (**Supplementary Figure 9**). This result indicates that *eur1-11* mutant is not defective in response to auxin. Therefore, the partial failure in rooting initiation under IAA treatment of *eur1-11* implies that the *EAR1*/*EUR1*-mediated excision UR was necessary to enhance AR formation, although auxin could induce AR formation independently. All these results support that leaf excision triggers an auxin-induced endogenous oscillation in gene expression that enhances root regeneration, which is mediated by EAR1/EUR1, a regulator of the excision UR (**Figure 5G**).

## Discussion

Here, we discover a UR and provide many evidences to support a link between an excision UR and AR formation in *Arabidopsis*. Promoter–luciferase analyses showed that the excision UR robustly appeared at petiole region in excised leaves (**Figure 1F**) where DNRR occurs (Chen et al., 2014) and was positively associated with DNRR (**Figure 2**). Transcriptomic analysis indicated that the excision UR reset expression patterns of many DNRR-associated genes (**Figures 3D–G** and **Supplementary Table 1**). Interestingly, two independent complementation groups (*EUR1* and *EUR2*) of the excision UR regulators were randomly isolated but simultaneously controlled AR formation (**Figure 4** and **Supplementary Figure 4**). Like other ultradian rhythms in gene expression, such as the root branching rhythm in *Arabidopsis* (Moreno-Risueno et al., 2010), segmentation and somitogenesis in *Drosophila* (Palmeirim et al., 1997), this excision UR is involved in a developmental process, DNRR. In addition, like root clock (Perez-Garcia et al., 2022) and segmentation clock (Pourquié, 2003), there might be a ultradian clock to regulate this excision UR, in which *EUR1* and *EUR2* play a role as core clock genes. However, the excision UR is not associated with a spatially periodic pattern of modular developmental. Instead, it is evoked *de novo* at the petiole region of excised leaves and is observed transiently after excision. Thus, the latent and transient excision UR has a unique oscillatory feature. DNRR is a highly complex process that involves regulatory networks that change over time and show three distinct phases (Xu, 2018; Jing et al., 2020). The time-frame of the excision UR overlapped with phase II (auxin accumulation) and phase III (cell fate transition) (**Figure 1E** and **Supplementary Table 1**). Therefore, cells undergo fate transition when the excision UR is robust. This timing is indicative of the role of the latent and transient excision UR in biological processes. In addition, expression of cell fate transition genes was altered in *eur1-11* mutants (**Figure 5C**). Rhythmic gene expression at the excision site may serve as a means of resetting and reprogramming gene expression to facilitate cell fate transition and later enhance AR formation. This would resemble the situation in lateral root development, in which oscillatory behaviour of some genes is associated with cell fate transition in response to lateral root initiation (Moreno-Risueno et al., 2010; Gala et al., 2021).

Robustness of the excision UR was affected by developmental stage of leaves and environmental signals such as light intensity, which also influence DNRR efficiency (**Figure 2**). As plants age, gradually increased transcription factors such as *SQUAMOSA PROMOTER BINDING PROTEIN-LIKE* (*SPL*) *2/10/11* and *ETHYLENE INSENSITIVE 3* (*EIN3*) repress root regeneration by inhibiting auxin biosynthesis and expression of cell fate transition genes, respectively (Li et al., 2020; Ye et al., 2020). Auxin, a major hormone in DNRR, was required for generation of the excision UR (**Figures 1J, K**) and also rescued the excision UR in aged leaves (**Figure 5E**). This indicates that the excision UR as well as DNRR are positively regulated by auxin which level is gradually decreased along with the age of leaves. Interestingly, treatment of young, excised leaves with exogenous auxin did not significantly affect the UR wavelet power (**Figure 1J**), suggesting that endogenous auxin levels were sufficient for UR generation in young leaves. Proper light intensity was required for optimal generation of the excision UR (**Figure 2D**). This may be caused by an imbalance in carbohydrate concentration, which is otherwise required for optimal DNRR (**Figures 2E, F**). Previous study showed that, in excised leaves, sucrose is required in the dark to regenerate ARs, but somewhat represses root regeneration in the light (Chen et al., 2014), suggesting that an appropriate amount of carbohydrate is necessary for optimal root regeneration as an energy source. Lower robustness of the excision UR in the dark (**Figure 2D**) might also be caused by depletion of energy which can be made by photosynthesis in the light. As only leaves can make enough energy source *via* photosynthesis in the light, leaf-specific occurrence of the excision UR (**Supplementary Figure 2**) supports this explanation. However, regeneration occurs frequently in nature in both plants and animals to recover lost or damaged tissues and organs (Stoick-Cooper et al., 2007; Xu and Huang, 2014). Therefore, it is worth checking whether a similar oscillatory mechanism might function to optimize regeneration in other species.

Leaf excision and subsequent DNRR processes are largely integrated by the interplay of several hormones, including early signaling by the wound hormone jasmonic acid followed by various auxin, cytokinin and ethylene (Lakehal and Bellini, 2019; Liu et al., 2022). This is consistent with the KEGG pathway analysis of the excision UR transcriptome (**Figure 3C**) as the excision UR is associated with DNRR. However, the role of ABA signaling components in DNRR has been rarely discussed to date. One of the regulators of the excision UR identified from genetic screening was EAR1/EUR1, previously known as a negative regulator of ABA signalling (Wang et al., 2018). Interestingly, EAR1/EUR1 is involved in canonical ABA responses, but the excision UR mediated by EAR1/EUR1 may be generated by a different molecular mechanism (**Figures 4H-K** and **Supplementary Figure 7**), which is positively regulated by auxin. ABA is generally considered as a negative regulator of AR formation (Lakehal and Bellini, 2019). Therefore, although EAR1/EUR1-mediated excision UR generation and root regeneration was decoupled from canonical ABA responses, EAR1/EUR1 may also regulate ABA signaling during DNRR by activation of the ABA co-receptor phosphatases that negatively regulate ABA signaling, and to evoke the excision UR at the excision site. Consistent with these, the expression of EAR1/EUR1 is activated at the excision site of petiole, as would be expected for the petiole excision site to be competent for cell fate transition and division. How is EAR1/EUR1 involved in the regulation of the excision UR? *EAR1/EUR1* encodes an uncharacterized protein which is mostly composed of intrinsically disordered domains and interacts with various proteins (Wang et al., 2018), suggesting that the excision UR might be based on complex regulatory networks of core components including EAR1/EUR1. Further studies to identify more components, such as other *eur* mutants or factors interacting with EAR1/EUR1, will improve our understanding of the regulatory mechanisms underlying the excision UR in DNRR.

## Conclusion

Biological rhythms are ubiquitous in most organisms and play critical roles for responses to environmental changes or developmental processes. However, unlike well-known circadian rhythm, origin and biological significance of ultradian rhythms (URs) remain opened questions and many biological processes which may be associated with URs have not been identified yet. Here, we discovered a new ~3-h UR in excised *Arabidopsis* leaves. Taking advantages of transcriptomic analysis and forward genetic screen, we found more than 4,000 oscillating genes involved in a range of biological processes and two key regulators (*EUR1* and *EUR2*) driving this oscillation. Our work provided a useful data source for further studies to investigate functions, regulatory mechanism and significance of URs. Our physiological experiments also indicated a close relationship between UR and *de novo* root regeneration (DNRR) in *Arabidopsis* leaves. Mutation of key UR regulators, *eur1* and *eur2*, both delayed rooting initiation, supporting that UR may be required to enhance DNRR, which increases fitness through vegetative propagation. Understanding the mechanisms that regulates the excision UR will facilitate effective vegetative propagation of plants and improve our fundamental understanding of explant regeneration.

## Data availability statement

The datasets presented in this study can be found in online repositories. The names of the repository/repositories and accession number(s) can be found in the article/**Supplementary Material**.

## Author contributions

Conceptualization: QV, KS, HN, SH. Methodology: QV, KS, SH. Formal analysis: KS, SH. Investigation: QV, SP, SH. Supervision: HN, SH. Writing – original draft: QV, KS, SH. Writing – review & editing: LX, HN, SH. All authors contributed to the article and approved the submitted version.

## Supplementary material

Supplementary Figure 1 Time-series analysis of CCA1, PRR7, CAB2 and ORE1 promoter activities. **(A)** Experimental setup used to measure luminescence in the leaves of transgenic Arabidopsis plants expressing Luciferase gene. **(B, C, F, G)** Activities of CCA1 **(B)**, PRR7 **(C)**, CAB2 **(F)** and ORE1 **(G)** promoters in excised Arabidopsis leaves at the indicated time points. LUC intensity was measured every 30 min under continuous white light conditions at 22°C. Each graph shows three representative samples (n = 24 leaves); at least three different experiments were performed with similar results. **(D, E, H, I)** Wavelet analyses of the activities of CCA1 **(D)**, PRR7 **(E)**, CAB2 **(H)** and ORE1 **(I)** promoters, based on LUC intensity. In each plot, the upper left and right panels show the circadian (CR) and ultradian (UR) rhythms, respectively; and the lower panels show the wavelet spectra. Each wavelet spectrum plots shows merged wavelet power plots of all samples with low transparency.

Supplementary Figure 2 The ultradian rhythm occurs in excised leaves. **(A, D, J)** Experimental setup used to measure luminescence in different tissues excised from transgenic Arabidopsis plants expressing ORE1::LUC. **(B, C, E-I, K-M)** ORE1 promoter activity in attached **(B)** and excised **(C)** 3rd and 4th rosette leaves of 21-day-old plants; in whole seedlings **(E)**, excised shoot apexes **(F)**, excised cotyledons **(G)**, excised hypocotyls **(H)** and excised roots **(I)** of 7-day-old seedlings; and in excised flowers **(K)**, excised cauline leaves **(L)** and excised stems **(M)** of 35-day-old bolted plants at the indicated time points. The graphs show three representative samples; at least three different experiments were performed with similar results. **(N)** Average wavelet powers of ultradian rhythms (UR) in various Arabidopsis samples quantified by wavelet analysis. In **(B, C, E-I, K-M)**, n = 24 tissue samples per experiment. In (n) centre line: median; bounds of box: 25th and 75th percentiles; whiskers: 1.5 × IQR from 25th and 75th percentiles. Statistical significance was determined by one-way analysis of variance (ANOVA) with Tukey’s *post hoc* test. Data points with different letters indicate statistically significant differences between groups (P < 0.01). Red line indicates the UR threshold.

Supplementary Figure 3 Comparison of oscillating genes with microarray data from the root clock and DNRR-associated genes. Venn diagram showing the number of genes overlapping between the different groups: excision UR genes, root clock genes involved in the production of prebranch sites, and DNRR-associated genes.

Supplementary Figure 4 Screening of eur mutant candidates and identification of EUR1 as EAR1, a negative regulator of ABA signaling. **(A)** ORE1 promoter activity in M38 (eur2) mutant candidate derived from EMS mutagenesis of ORE1::LUC transgenic Arabidopsis seeds. The graph shows three representative samples. **(B, D)** Comparison of wild-type and M38 leaves showing average wavelet powers of the excision UR **(B)** and rooting rates **(D)**. In **(B)**, n = 24 leaves; centre line: median; bounds of box: 25th and 75th percentiles; whiskers: 1.5 × IQR from 25th and 75th percentiles. The two-tailed t-test was used to determine statistically significant differences between wild-type and M38 plants (*P ≤ 0.05; **P≤ 0.01). In **(D)**, data are means ± s.e.m. from three independent replicates). **(C)** Genetic complementation analysis of eur mutant candidates. Data indicate the rescue of the excision UR phenotype following reciprocal crosses between candidates and their parental genotype (Col/ORE1::LUC), or after crosses between mutant candidates (M21, 23, 38 and 83). +: rescued excision UR; -: no rescue of excision UR; ×: no crossing. **(E)** Whole genome sequencing of eur1-11 (M21) and eur1-13 (M83). Data indicate the genome-wide distribution of variants on each chromosome, along with the positions and allele frequencies of SNPs detected in mutant candidates. The ratio represents the allele frequency; gene names show candidates with GC to AT SNPs that can lead to amino acid changes. The gene common to both eur1-11 (M21) and eur1-13 (M83) is labelled with a red asterisk. **(F)** Amino acid alignment of EAR1 sequences from wild-type, eur1-11 (M21), eur1-12 (M23), and eur1-13 (M83) plants.

Supplementary Figure 5 Complementation of eur1-11 rescued the impaired excision UR phenotype and delayed AR initiation. **(A)** Average wavelet powers of the excision UR in WT, eur1-11 and two complementation lines (COM-9 and COM-24) expressing EUR1/EAR1 (EUR1::EUR1-GFP) in the eur1-11 mutant background (n = 24 leaves). Centre line: median; bounds of box: 25th and 75th percentiles; whiskers: 1.5 × IQR from 25th and 75th percentiles. Statistical significance was determined by one-way analysis of variance (ANOVA) with Tukey’s *post hoc* test. Data points with different letters indicate statistically significant differences between groups (P < 0.01). **(B)** Rooting rates of WT, eur1-11, COM-9 and COM-24 plants. Data are means ± s.e.m. from three independent replicates.

Supplementary Figure 6 The excision UR was gone in ear1-1 mutant. Average wavelet powers of PIN3, AFB2 and KMD1 promoter excision UR in Col and ear1-1 mutant (n = 24 leaves). Centre line: median; bounds of box: 25th and 75th percentiles; whiskers: 1.5 × IQR from 25th and 75th percentiles. Two-tailed t-test was used between Col and ear1-1 for each transgenic line.

Supplementary Figure 7 EAR1^141-287^ fragment is not enough to recover the excision UR and DNRR efficiency in *eur1-11* mutant. (A-B) Representative images of seed germination greening ratio of *Col/ORE1::LUC*, *eur1-11* and *EAR1::EAR1^141-287^-FLAG* transgenic lines on *eur1-11* mutant background under ABA treatment. 30 seeds per line were sowed with three replicates. At least two independent experiments were done with similar results. Thirty-five T1 *EAR1::EAR1^141-287^-FLAG* transgenic plants were selected on 20 mg/ml DL-phosphinothricin (PPT)- containing medium. T2 lines were then grown under PPT-containing medium to confirm the number of copies. Here, four random T2 lines were selected for experiments with two T2 lines (EAR1^141-287^ T2_2 and T2_4) containing multiple copies of transgene (almost seeds among 40 sowed seeds germinated and survived in PPT selection medium) and two other lines (EAR1^141-287^ T2_10 and T2_18) containing one copy of transgene (surviving seedlings: dead seedlings ~ 3:1 ratio). Scale bar: 1cm. (C) Germination greening ratio in (A-B) (n=3). Two-tailed t-test was used between *Col/ORE1::LUC* and *eur1-11* or *EAR1::EAR1^141-287^-FLAG* transgenic lines (* *p* < 0.05). (D) Average wavelet power of *ORE1* promoter excision UR in *Col/ORE1::LUC*, *eur1-11*, *EAR1::EAR1^141-287^-FLAG* transgenic plants on *eur1-11* mutant background (*n* = 35 leaves from 35 T1 plants) and four T2 lines (*n* = 24 leaves/line). (E) Rooting rates of *Col/ORE1::LUC*, *eur1-11*, four T2 lines of *EAR1::EAR1^141-287^-FLAG* transgenic plants. Data are means ± s.e.m. (*n* = 12).

Supplementary Figure 8 Cluster analysis of genes differentially expressed between the petiole regions of wild-type and eur1-11 mutant leaves at 0, 24, 48, 72 and 96 h after excision.

Supplementary Figure 9 eur1-11 is not an auxin resistant mutant. **(A)** Representative images of Col/ORE1::LUC, eur1-11 and axr1-3 seedlings without IAA treatment or with 0.2µM IAA treatment. Scale bar: 1cm. **(B)** Quantified data in **(A)**. Data are means ± s.e.m. from at least 10 independent seedlings per line. Two-tailed t-test was used between Col/ORE1::LUC and eur1-11 or axr1-3 mutants (ns: non-significance, *** p ≤ 0.001). Seeds of Col/ORE1::LUC, eur1-11 and axr1-3 were sowed on vertical half strength MS medium plates under long day (16h light/8h dark). After 5 days, seedlings were transferred to half strength MS medium plates containing different concentrations of IAA with marked root tip positions and grown for additional 3 days. The plates were then scanned by HP Scanjet 8300 and primary root elongation was measured by ImageJ program.
