# Supplementary figures and images for: An auxin-mediated ultradian rhythm positively influences root regeneration via EAR1/EUR1 in Arabidopsis
Source: Front Plant Sci. 2023 Jun 7;14:1136445. doi: 10.3389/fpls.2023.1136445 (PMC10282773; doi:10.3389/fpls.2023.1136445)

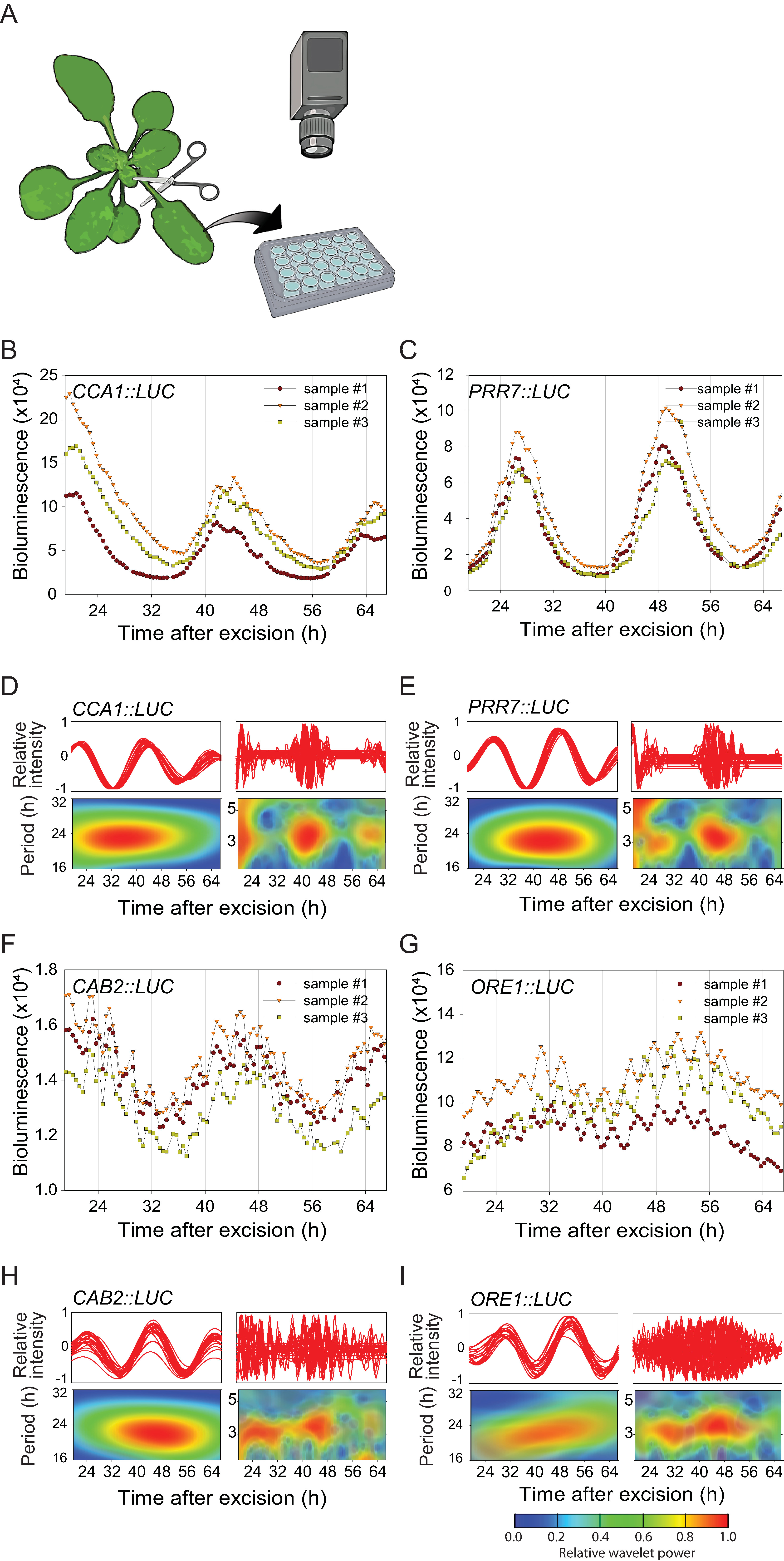

Supplement: Supplementary Figure 1 — Time-series analysis of CCA1, PRR7, CAB2 and ORE1 promoter activities. (A) Experimental setup used to measure luminescence in the leaves of transgenic Arabidopsis plants expressing Luciferase gene. (B, C, F, G) Activities of CCA1 (B), PRR7 (C), CAB2 (F) and ORE1 (G) promoters in excised Arabidopsis leaves at the indicated time points. LUC intensity was measured every 30 min under continuous white light conditions at 22°C. Each graph shows three representative samples (n = 24 leaves); at least three different experiments were performed with similar results. (D, E, H, I) Wavelet analyses of the activities of CCA1 (D), PRR7 (E), CAB2 (H) and ORE1 (I) promoters, based on LUC intensity. In each plot, the upper left and right panels show the circadian (CR) and ultradian (UR) rhythms, respectively; and the lower panels show the wavelet spectra. Each wavelet spectrum plots shows merged wavelet power plots of all samples with low transparency. [file Image_1.jpeg]

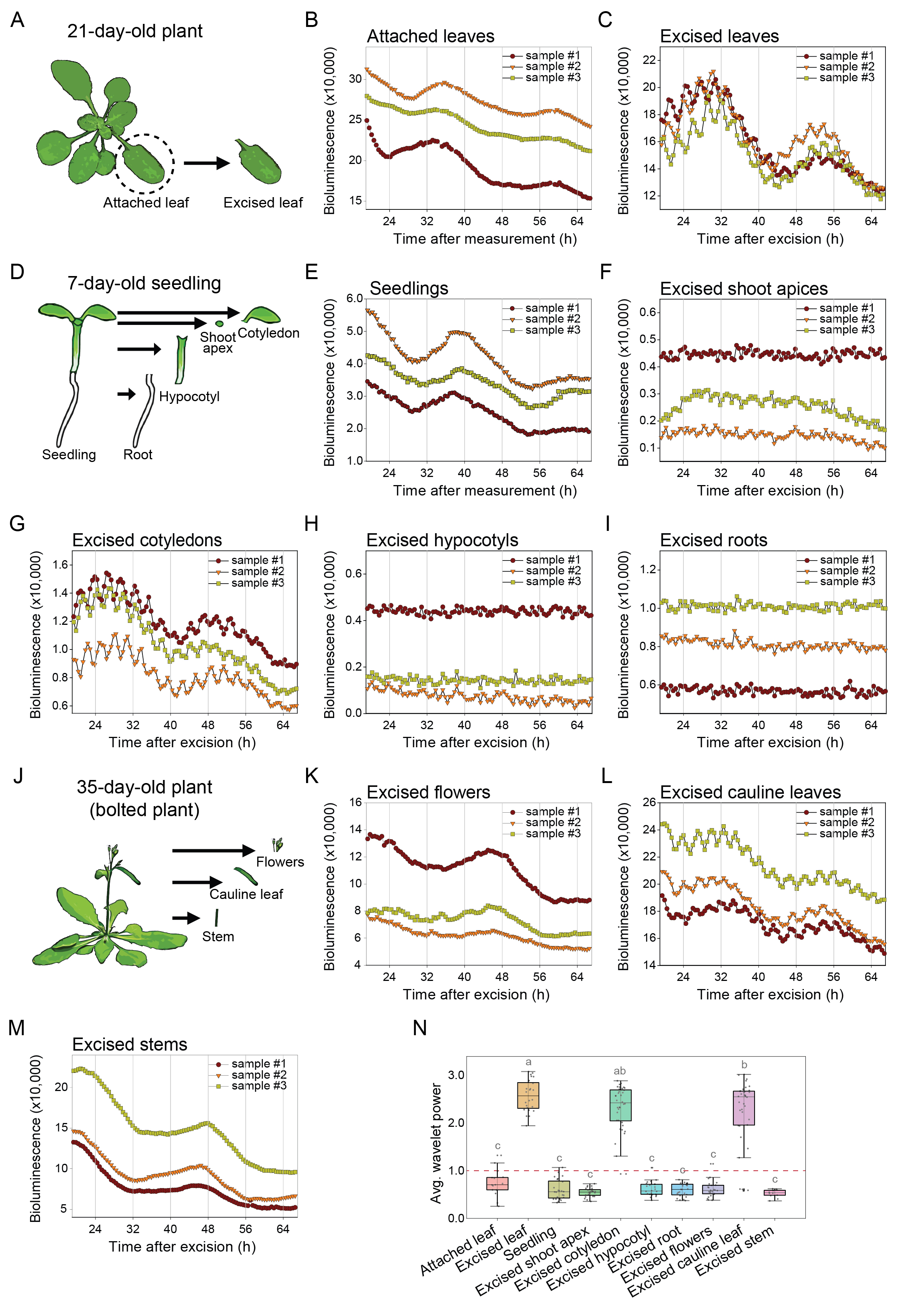

Supplement: Supplementary Figure 2 — The ultradian rhythm occurs in excised leaves. (A, D, J) Experimental setup used to measure luminescence in different tissues excised from transgenic Arabidopsis plants expressing ORE1::LUC. (B, C, E-I, K-M) ORE1 promoter activity in attached (B) and excised (C) 3rd and 4th rosette leaves of 21-day-old plants; in whole seedlings (E), excised shoot apexes (F), excised cotyledons (G), excised hypocotyls (H) and excised roots (I) of 7-day-old seedlings; and in excised flowers (K), excised cauline leaves (L) and excised stems (M) of 35-day-old bolted plants at the indicated time points. The graphs show three representative samples; at least three different experiments were performed with similar results. (N) Average wavelet powers of ultradian rhythms (UR) in various Arabidopsis samples quantified by wavelet analysis. In (B, C, E-I, K-M), n = 24 tissue samples per experiment. In (n) centre line: median; bounds of box: 25th and 75th percentiles; whiskers: 1.5 × IQR from 25th and 75th percentiles. Statistical significance was determined by one-way analysis of variance (ANOVA) with Tukey’s post hoc test. Data points with different letters indicate statistically significant differences between groups (P < 0.01). Red line indicates the UR threshold. [file Image_2.jpeg]

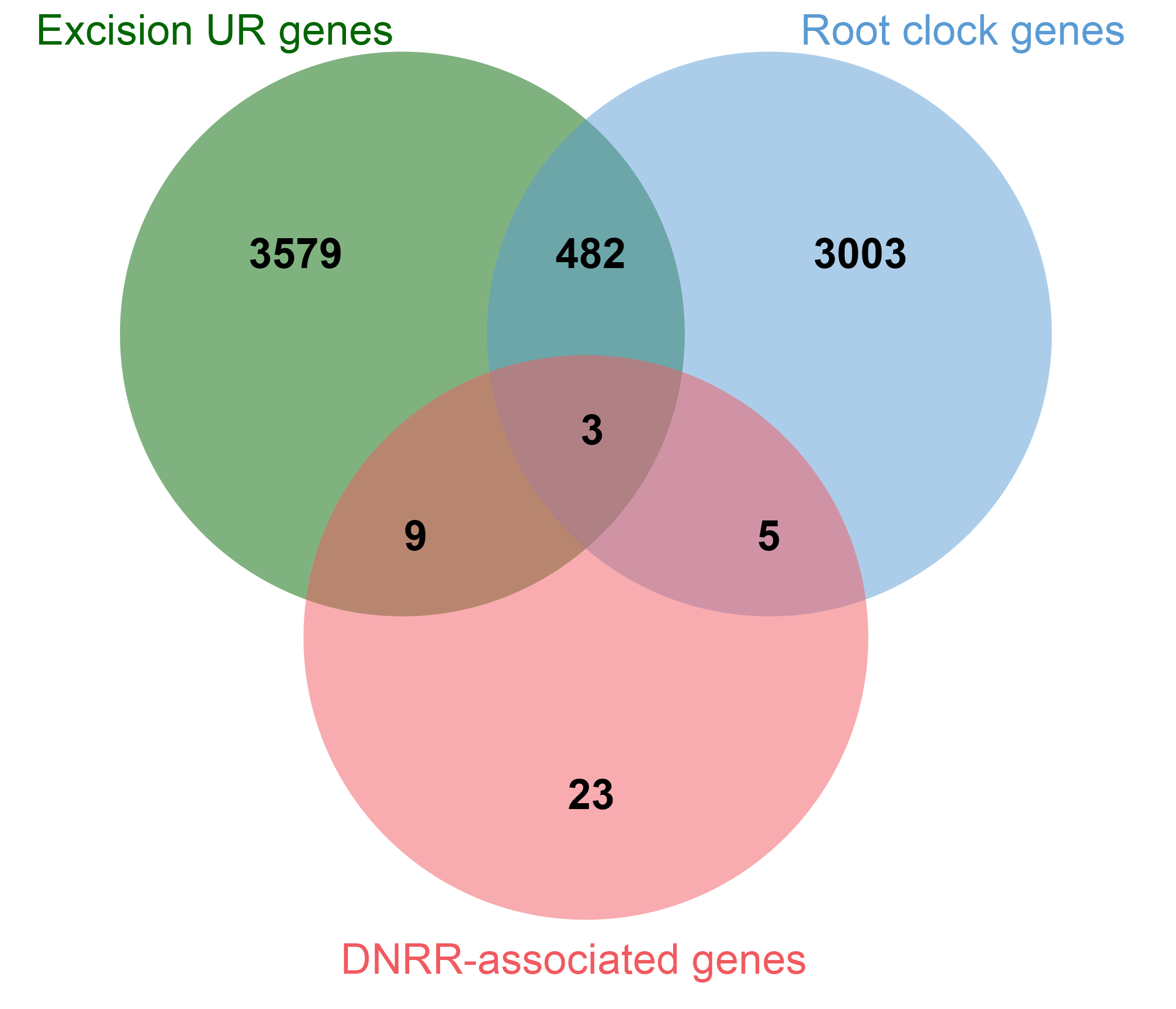

Supplement: Supplementary Figure 3 — Comparison of oscillating genes with microarray data from the root clock and DNRR-associated genes. Venn diagram showing the number of genes overlapping between the different groups: excision UR genes, root clock genes involved in the production of prebranch sites, and DNRR-associated genes. [file Image_3.jpeg]

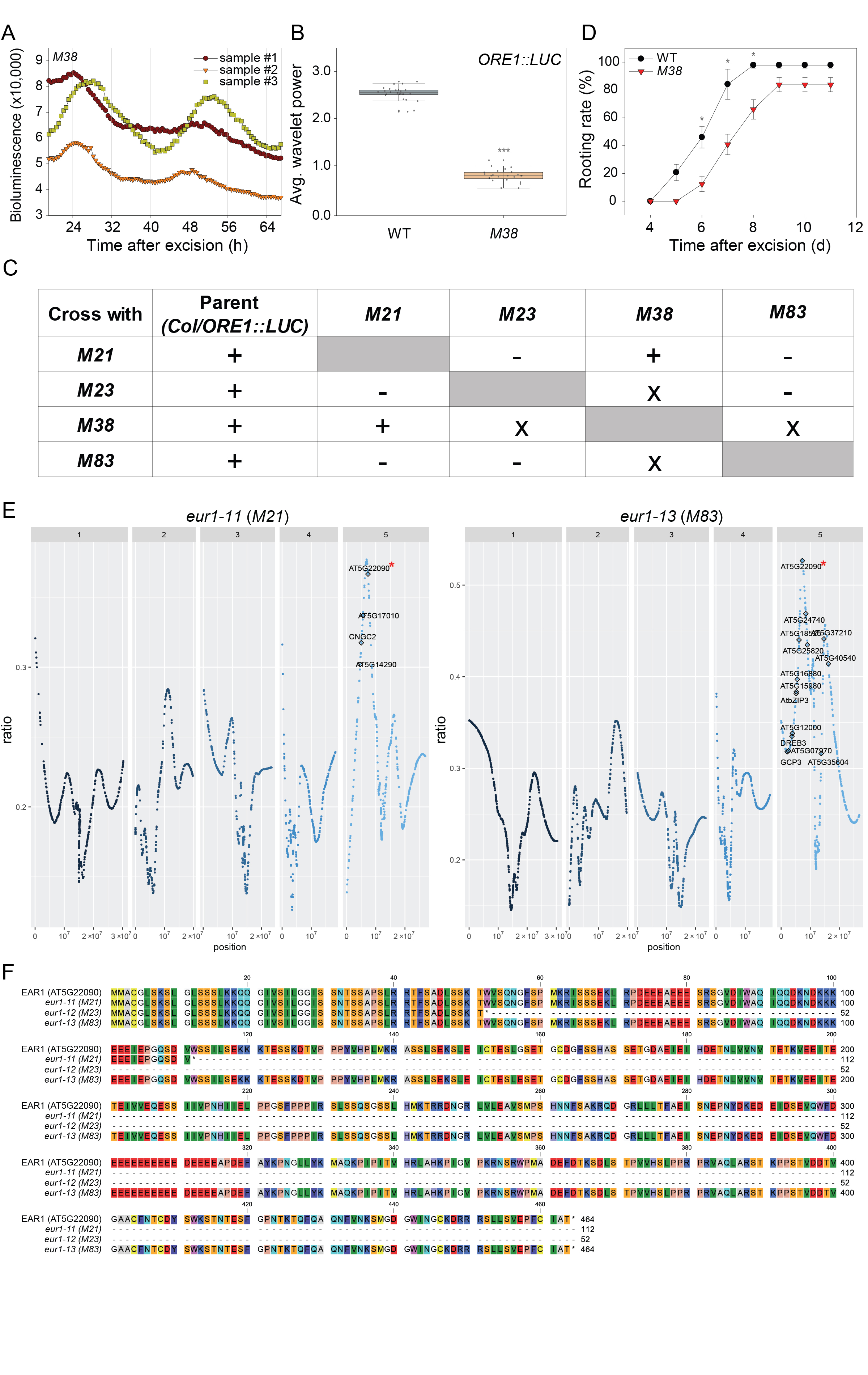

Supplement: Supplementary Figure 4 — Screening of eur mutant candidates and identification of EUR1 as EAR1, a negative regulator of ABA signaling. (A) ORE1 promoter activity in M38 (eur2) mutant candidate derived from EMS mutagenesis of ORE1::LUC transgenic Arabidopsis seeds. The graph shows three representative samples. (B, D) Comparison of wild-type and M38 leaves showing average wavelet powers of the excision UR (B) and rooting rates (D). In (B), n = 24 leaves; centre line: median; bounds of box: 25th and 75th percentiles; whiskers: 1.5 × IQR from 25th and 75th percentiles. The two-tailed t-test was used to determine statistically significant differences between wild-type and M38 plants (*P ≤ 0.05; **P≤ 0.01). In (D), data are means ± s.e.m. from three independent replicates). (C) Genetic complementation analysis of eur mutant candidates. Data indicate the rescue of the excision UR phenotype following reciprocal crosses between candidates and their parental genotype (Col/ORE1::LUC), or after crosses between mutant candidates (M21, 23, 38 and 83). +: rescued excision UR; -: no rescue of excision UR; ×: no crossing. (E) Whole genome sequencing of eur1-11 (M21) and eur1-13 (M83). Data indicate the genome-wide distribution of variants on each chromosome, along with the positions and allele frequencies of SNPs detected in mutant candidates. The ratio represents the allele frequency; gene names show candidates with GC to AT SNPs that can lead to amino acid changes. The gene common to both eur1-11 (M21) and eur1-13 (M83) is labelled with a red asterisk. (F) Amino acid alignment of EAR1 sequences from wild-type, eur1-11 (M21), eur1-12 (M23), and eur1-13 (M83) plants. [file Image_4.jpeg]

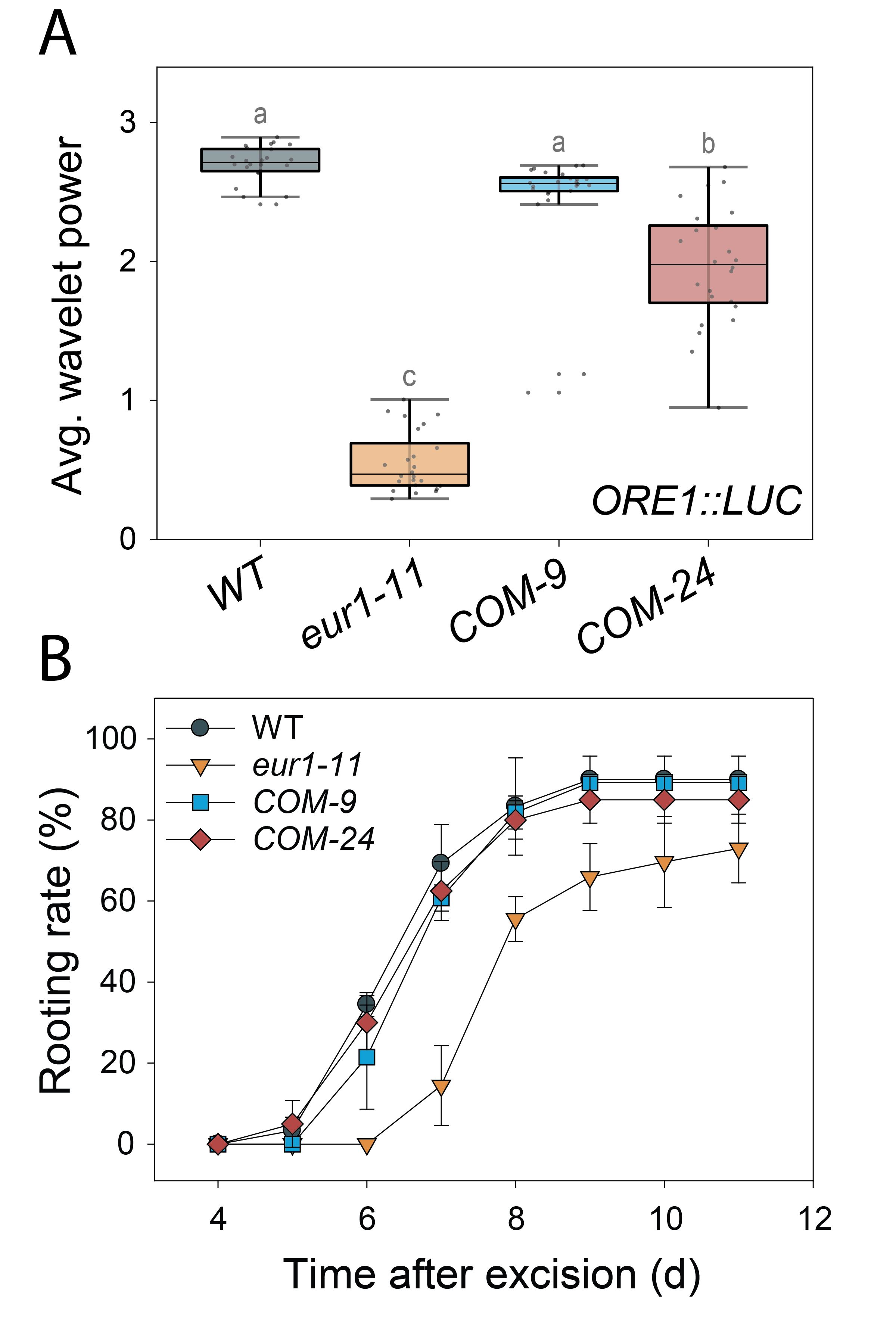

Supplement: Supplementary Figure 5 — Complementation of eur1-11 rescued the impaired excision UR phenotype and delayed AR initiation. (A) Average wavelet powers of the excision UR in WT, eur1-11 and two complementation lines (COM-9 and COM-24) expressing EUR1/EAR1 (EUR1::EUR1-GFP) in the eur1-11 mutant background (n = 24 leaves). Centre line: median; bounds of box: 25th and 75th percentiles; whiskers: 1.5 × IQR from 25th and 75th percentiles. Statistical significance was determined by one-way analysis of variance (ANOVA) with Tukey’s post hoc test. Data points with different letters indicate statistically significant differences between groups (P < 0.01). (B) Rooting rates of WT, eur1-11, COM-9 and COM-24 plants. Data are means ± s.e.m. from three independent replicates. [file Image_5.jpeg]

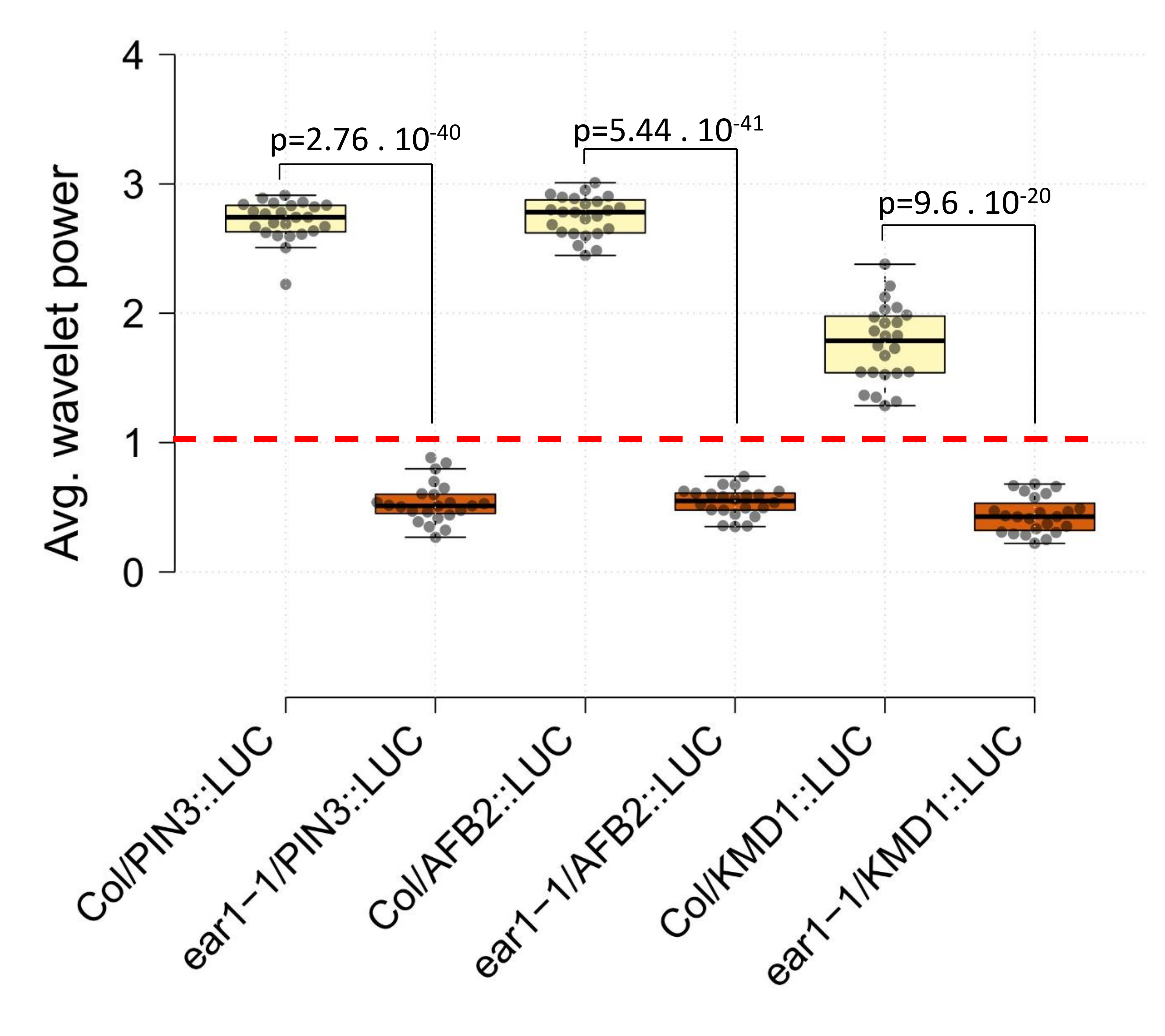

Supplement: Supplementary Figure 6 — The excision UR was gone in ear1-1 mutant. Average wavelet powers of PIN3, AFB2 and KMD1 promoter excision UR in Col and ear1-1 mutant (n = 24 leaves). Centre line: median; bounds of box: 25th and 75th percentiles; whiskers: 1.5 × IQR from 25th and 75th percentiles. Two-tailed t-test was used between Col and ear1-1 for each transgenic line. [file Image_6.jpeg]

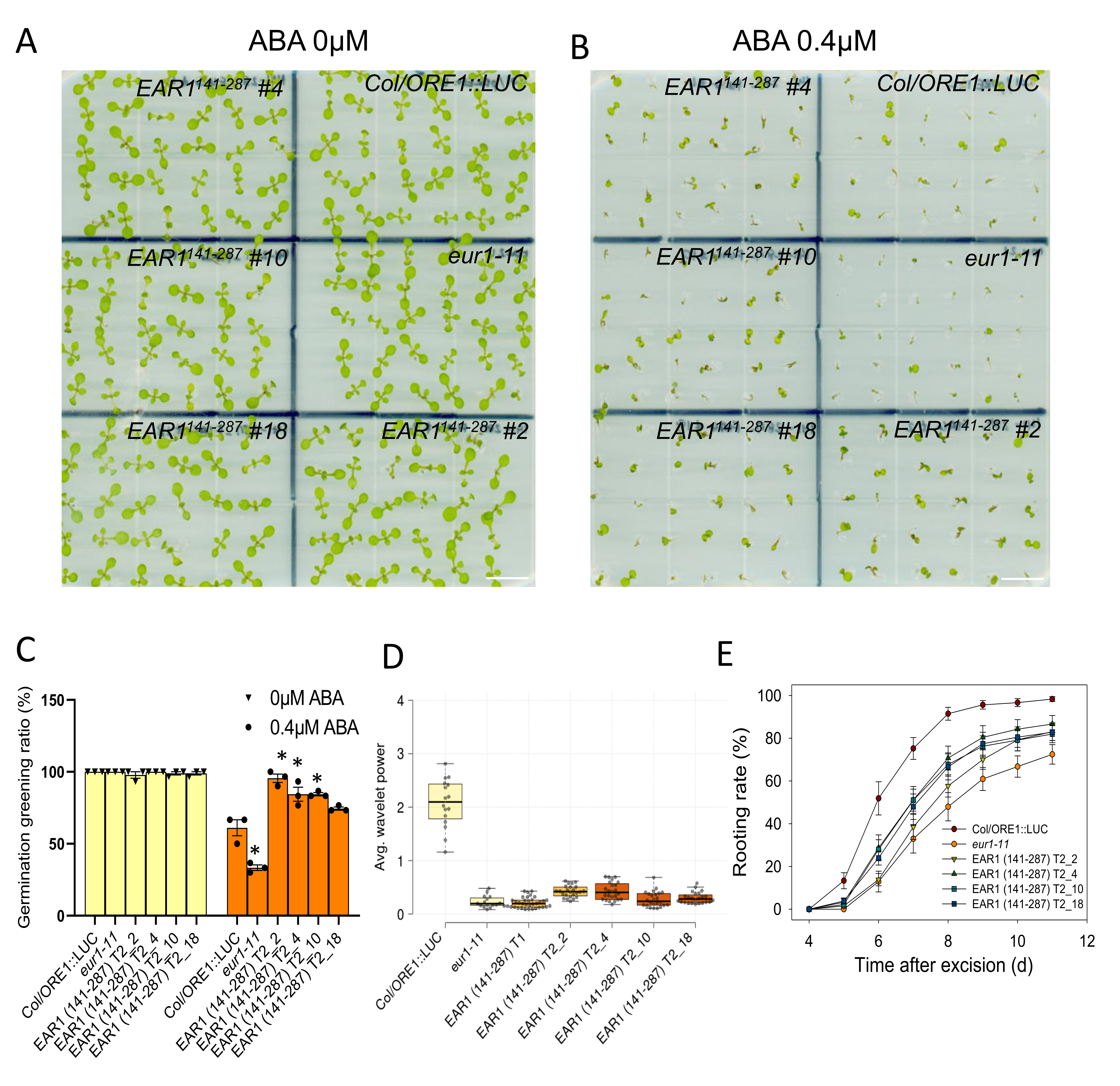

Supplement: Supplementary Figure 7 — EAR1141-287 fragment is not enough to recover the excision UR and DNRR efficiency in eur1-11 mutant. (A-B) Representative images of seed germination greening ratio of Col/ORE1::LUC, eur1-11 and EAR1::EAR1141-287-FLAG transgenic lines on eur1-11 mutant background under ABA treatment. 30 seeds per line were sowed with three replicates. At least two independent experiments were done with similar results. Thirty-five T1 EAR1::EAR1141-287-FLAG transgenic plants were selected on 20 mg/ml DL-phosphinothricin (PPT)- containing medium. T2 lines were then grown under PPT-containing medium to confirm the number of copies. Here, four random T2 lines were selected for experiments with two T2 lines (EAR1141-287 T2_2 and T2_4) containing multiple copies of transgene (almost seeds among 40 sowed seeds germinated and survived in PPT selection medium) and two other lines (EAR1141-287 T2_10 and T2_18) containing one copy of transgene (surviving seedlings: dead seedlings ~ 3:1 ratio). Scale bar: 1cm. (C) Germination greening ratio in (A-B) (n=3). Two-tailed t-test was used between Col/ORE1::LUC and eur1-11 or EAR1::EAR1141-287-FLAG transgenic lines (* p < 0.05). (D) Average wavelet power of ORE1 promoter excision UR in Col/ORE1::LUC, eur1-11, EAR1::EAR1141-287-FLAG transgenic plants on eur1-11 mutant background (n = 35 leaves from 35 T1 plants) and four T2 lines (n = 24 leaves/line). (E) Rooting rates of Col/ORE1::LUC, eur1-11, four T2 lines of EAR1::EAR1141-287-FLAG transgenic plants. Data are means ± s.e.m. (n = 12). [file Image_7.jpeg]

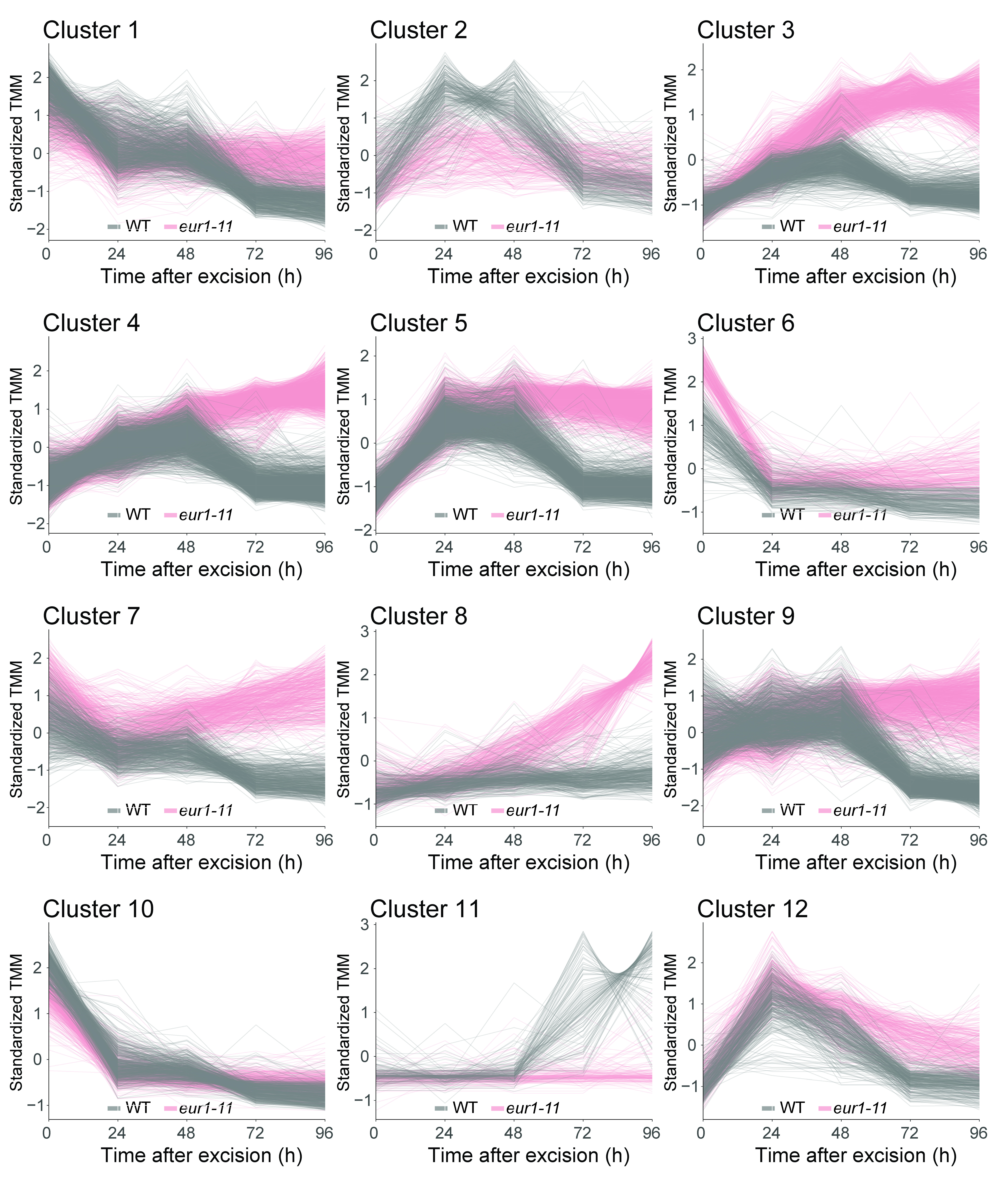

Supplement: Supplementary Figure 8 — Cluster analysis of genes differentially expressed between the petiole regions of wild-type and eur1-11 mutant leaves at 0, 24, 48, 72 and 96 h after excision. [file Image_8.jpeg]

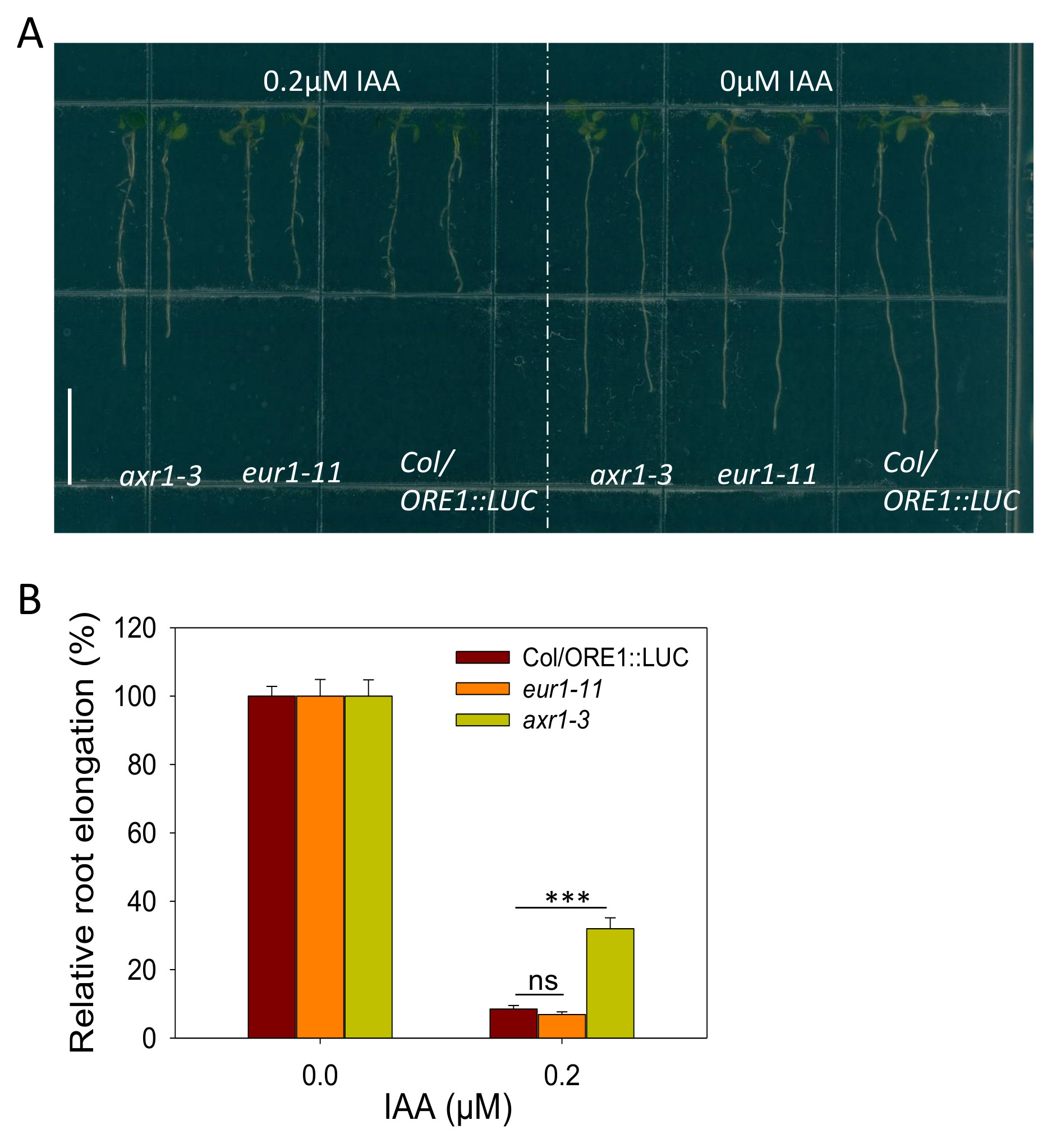

Supplement: Supplementary Figure 9 — eur1-11 is not an auxin resistant mutant. (A) Representative images of Col/ORE1::LUC, eur1-11 and axr1-3 seedlings without IAA treatment or with 0.2µM IAA treatment. Scale bar: 1cm. (B) Quantified data in (A). Data are means ± s.e.m. from at least 10 independent seedlings per line. Two-tailed t-test was used between Col/ORE1::LUC and eur1-11 or axr1-3 mutants (ns: non-significance, *** p ≤ 0.001). Seeds of Col/ORE1::LUC, eur1-11 and axr1-3 were sowed on vertical half strength MS medium plates under long day (16h light/8h dark). After 5 days, seedlings were transferred to half strength MS medium plates containing different concentrations of IAA with marked root tip positions and grown for additional 3 days. The plates were then scanned by HP Scanjet 8300 and primary root elongation was measured by ImageJ program. [file Image_9.jpeg]
